# Extracellular vesicles originating from melanoma cells promote dysregulation in haematopoiesis as a component of cancer immunoediting

**DOI:** 10.1002/jev2.12471

**Published:** 2024-06-29

**Authors:** Doste R. Mamand, Safa Bazaz, Dara K. Mohammad, Xiuming Liang, Svetlana Pavlova, Carsten Mim, Susanne Gabrielsson, Joel Z. Nordin, Oscar P. B. Wiklander, Manuchehr Abedi‐Valugerdi, Samir EL‐Andaloussi

**Affiliations:** ^1^ Biomolecular and Cellular Medicine (BCM), Department of Laboratory Medicine Karolinska Institutet Huddinge Sweden; ^2^ Department of Cellular Therapy and Allogeneic Stem Cell Transplantation (CAST) Karolinska University Hospital Huddinge and Karolinska Comprehensive Cancer Center Stockholm Sweden; ^3^ Center for Hematology and Regenerative Medicine (HERM), Department of Medicine Huddinge Karolinska Institutet Stockholm Sweden; ^4^ College of Agricultural Engineering Sciences Salahaddin University‐Erbil Erbil Iraq; ^5^ Department of Protein Science KTH Royal Institute of Technology Stockholm Sweden; ^6^ Division of Immunology and Allergy, Department of Medicine Solna Karolinska Institutet Solna Sweden; ^7^ Department of Clinical Immunology and Transfusion Medicine (KITM) Karolinska University Hospital Stockholm Sweden; ^8^ Breast Center, Karolinska Comprehensive Cancer Center, Karolinska University Hospital, Stockholm, Sweden Karolinska Institute Stockholm Sweden; ^9^ Karolinska ATMP Center, ANA Futura, Huddinge, Sweden Karolinska Institute Stockholm Sweden

**Keywords:** angiogenesis factors, erythroid progenitor cells (EPCs), immune escape, immunosuppression, MDSCs, tEVs, vascular endothelial growth factor

## Abstract

Haematopoiesis dysregulation with the presence of immature myeloid and erythroid immunosuppressive cells are key characteristics of the immune escape phase of tumour development. Here, the role of in vitro generated B16F10 tumour cell‐derived extracellular vesicles (tEVs) as indirect cellular communicators, participating in tumour‐induced dysregulation of haematopoiesis, was explored. The isolated tEVs displayed features of small EVs with a size range of 100–200 nm, expressed the common EV markers CD63, CD9, and Alix, and had a spherical shape with a lipid bilayer membrane. Proteomic profiling revealed significant levels of angiogenic factors, particularly vascular endothelial growth factor (VEGF), osteopontin, and tissue factor, associated with the tEVs. Systemic administration of these tEVs in syngeneic mice induced splenomegaly and disrupted haematopoiesis, leading to extramedullary haematopoiesis, expansion of splenic immature erythroid progenitors, reduced bone marrow cellularity, medullary expansion of granulocytic myeloid suppressor cells, and the development of anaemia. These effects closely mirrored those observed in tumour‐bearing mice and were not seen after heat inactivating the tEVs. In vitro studies demonstrated that tEVs independently induced the expansion of bone marrow granulocytic myeloid suppressor cells and B cells while reducing the frequency of cells in the erythropoietic lineage. These effects of tEVs were significantly abrogated by the blockade of VEGF or heat inactivation. Our findings underscore the important role of tEVs in dysregulating haematopoiesis during the immune escape phase of cancer immunoediting, suggesting their potential as targets for addressing immune evasion and reinstating normal hematopoietic processes.

## 1 INTRODUCTION

Cellular communication plays a fundamental role in the development of cancer, enabling tumour cells to reprogram the functionality of the neighbouring or distantly located stromal and immune cells (Chiodoni et al., 2019; Dominiak et al., 2020). While reprograming of stromal cells mainly promotes events that are associated with tumour angiogenesis, proliferation, invasion and metastasis, reprograming of immune cells can specifically foster immune escape (Chiodoni et al., 2019; Dominiak et al., 2020). As the terminal phase of cancer immunoediting (Kim et al., 2007; Mittal et al., 2014; Schreiber et al., 2011; Smyth et al., 2006), immune escape has been considered as a hallmark characteristic of cancer, that is, it provides conditions for the malignant cells to not only avoid the immune recognition, but also induce a general immunosuppressive status in the host (Kim et al., 2007; Mittal et al., 2014; Schreiber et al., 2011). To avoid immune recognition, tumour cells may receive communication signals from the immune cells to either down‐regulate the expression of MHC class I molecules or up‐regulate the expression of immunoinhibitory molecules such as programmed death‐ligand 1 (PD‐L1)) (Ju et al., 2020; Mittal et al., 2014; Schreiber et al., 2011; Smyth et al., 2006). In contrast to evading immune recognition, the induction of an immunosuppressive state mainly requires communicative signals provided by tumour cells that predominantly causes a dysregulated haematopoiesis, which in turn leads to massive production of immunosuppressive cells such as myeloid‐derived immunosuppressive cells in the bone marrow (BM) and erythroid progenitor cells (EPCs) in the spleen (Grzywa et al., 2021; Kamran et al., 2018; Krishnamoorthy et al., 2021; Mittal et al., 2014; Schreiber et al., 2011). As a result, these immunoregulatory cells produce substantial amounts of different immunosuppressive mediators (e.g., interleukin 10 (IL‐10), transforming growth factor beta (TGF‐b), arginases, and indoleamine‐pyrrole 2,3‐dioxygenase (IDO) that eventually lead to the suppression of tumour‐specific immune responses (Grzywa et al., 2021; Krishnamoorthy et al., 2021; Mittal et al., 2014; Schreiber et al., 2011; Smyth et al., 2006).

The exact mechanism(s) that governs the dysregulation of haematopoiesis during the immune escape phase remains elusive and requires further investigation. Nevertheless, to enhance our comprehension of the underlying mechanisms, we suggest that immune‐evading cancer cells possess the capacity to disrupt haematopoiesis by releasing immune‐haematopoiesis‐altering factors, which take the form of free mediators and/or extracellular vesicles (EVs). In the context of EVs, these subcellular organelles have garnered significant attention as pivotal agents in cell‐to‐cell communication over the past decade (Tao & Guo, 2020; Xu et al., 2018; Zhang et al., 2021). EVs are small, highly heterogenous membranous particles secreted by various mammalian and non‐mammalian cell types and have been found in essentially all body fluids (Witwer et al., 2013). Depending on their mechanisms of formation and sizes, EVs can be categorized as three major classes: exosomes, microvesicles (MVs), and apoptotic bodies (Rädler et al., 2023; Xu et al., 2018). Among these EV classes, only exosomes and MVs are released from living cells and known to have cell to cell communication functions in various physiological and pathophysiological settings (Gurung et al., 2021; Roudi et al., 2023; Wiklander et al., 2019). Exosomes with average sizes of 30–150 nm are produced inside the cells, where a series of pathways including endocytosis, formation of intraluminal vesicles, and development of multivesicular bodies (MVBs), are engaged (Gurung et al., 2021; Rädler et al., 2023; Xu et al., 2018). In contrast, MVs with sizes ranging from 50 to 1000 nm are produced through a budding/blebbing process which involves cell membrane reorganization, and repositioning of phosphatidylserine to the outer leaflet of the cell membrane (Gurung et al., 2021; Xu et al., 2018). With respect to their compositions, EVs as membranous particles can display different transmembrane proteins and lipids, including tetraspanins (CD9, CD63 and CD81), adhesion molecules (ICAM‐1 and integrins), antigen presenting molecules (MHC Classes I and II), soluble secreted proteins (cytokine growth and growth factors), signalling receptors, glycoproteins and lipids (cholesterol, ceramide and phosphatidylserine) (Gurung et al., 2021; Wen et al., 2017; Xu et al., 2018). Moreover, their lumen can be loaded with various cytoskeletal proteins (actin and cofilin), genetic materials (e.g. mRNA, miRNA, tRNA and DNA), heat shock proteins (hsp90, hsp84), enzymes (GAPDH and ATPase) and MVB‐associated proteins (Alix and TSG101) (Gurung et al., 2021; Wen et al., 2017; Xu et al., 2018).

Tumour derived EVs (tEVs) have been implicated in different facets of tumour‐induced immunosuppression, such as inactivation of NK and T cell anti‐tumour activities, inhibition of dendritic cell maturation, induction of MDSC expansion, and macrophage polarization (Tao & Guo, 2020). However, since tumours are considered as complex tissues consisting of not only proliferating cancer cells, but also different distinct cell types known as stromal cells (Hanahan & Weinberg, 2011), little is known as to what extent tEVs contribute to tumour‐induced dysregulation of haematopoiesis and immunosuppression. This study was designed to shed further light on this issue by employing EVs derived from B16F10 melanoma cells as a model system.

## 2 MATERIALS AND METHODS

### 2.1 Chemicals and reagents

Dulbecco's Modified Eagle's Medium (DMEM) (Gibco Life Technology), Opti‐MEM (Gibco Life Technology), Dulbecco's Phosphate Buffer Saline (DPBS) (Gibco Life Technology), foetal bovine serum, sodium pyruvate, L‐glutamine, 2‐mercaptoethanol, trypsin/EDTA, and 100× Antibiotic Antimycotic were all obtained from (Gibco Life Technology).

### 2.2 Cell culture

B16F10 melanoma cell line (B16F10 cells were engineered to express the luciferase firefly gene, luc2 (PerkinElmer, MA, USA) enabling in vitro and in vivo bioluminescent detection for monitoring tumour status) was purchased from American Type Culture Collection (ATCC) as described previously (Lennaárd et al., 2021). They were cultured in DMEM medium supplemented with 10% (FBS), 100 units/mL antimycotic, and 100 µg/mL antibiotic. The cell cultures were incubated at 37°C under a humidified atmosphere containing 5% CO_2_. The cells were passaged (the maximum passages were 16) every 3–4 days, routinely.

### 2.3 Harvesting cell culture media from primary skin cells

Fibroblasts were isolated according to the published protocol: https://dx.doi.org/10.3791/53565. with some minor changes. Shortly after, mice were euthanized by cervical dislocation according to the ethical permit described below. Ears from 2 C57Bl6/J mice were cut (∼1 cm), and hair was removed. Using scissors, ears were cut into small pieces (∼3 mm), transferred to the Eppendorf tubes prefilled with the collagenase D‐pronase mixture for digestion, and left on the shaker at 300 RPM for 90 min at 37°C. After incubation, the cell suspension was ground through a 70 µm cell strainer into the complete medium of RPMI 1640 with Glutamax, 10% FBC, 50 µM Mercaptoethanol, and 1% A/A. Further, cells were washed twice in complete media with a centrifugation step for 7 min at ∼580 × *g* at RT. After washing, 10 mL of the cells’ suspension was divided into two 100‐mm cell culture dishes. Cells were incubated at 37°C in a humidified 5% CO_2_ incubator. On the third day, the media was removed, cells were washed with sterile PBS, and new media was added. On day 6, cells were subcultured. For the experiment, 500,000 cells were seeded into 100‐mm dishes. After 48 h, the media was removed, cells were washed, and 5 mL of Opti‐mem media with 1% A/A was added to the plates.

### 2.4 Generating B16F10 stably expressing CD63‐mNG tEVs

Lentiviral supernatants were generated using the methods outlined in a prior study (reference 17). In brief, HEK‐293T cells were transfected at the same time with the p2CL9IPw5 construct, which encoded CD63 tagged to mNG, the pCD/NL‐BH helper construct, and the pcoPE human foamy virus envelope construct. This transfection was carried out using the transfection reagent PEI. The human CMV immediate‐early gene enhancer/promoter's gene expression was sped up 18 h after transfection by giving the cells 10 mM sodium butyrate (Sigma‐Aldrich) for 7 h. Following this, fresh medium was given to the cells, and the supernatant was collected after a total of 22 h. The viral particles were subsequently centrifuged at 25,000 × *g* for 90 min at 4°C. After removing the supernatant, the pellet was resuspended in 1 mL of Iscove's Modified Dulbecco's Media with 20% FBS and 1% antibiotic antimycotic. The viruses were stored at a temperature of −80°C until they were used. The B16F10 cells were subsequently transduced with the virus and cultured. They were then subjected to puromycin selection for at least five rounds of passage to generate stable cell lines.

### 2.5 Cell culture for the preparation of tEVs

B16F10 cells at the concentration of 10 million cells in 20 mL per Petri dish, 150 × 20 mm (SARSTEDT AG & Co. KG, Germany) were grown in DMEM as described above. After 24 h, the culture medium was changed to Opti‐MEM (serum‐free medium). Forty‐eight hours later, the conditioned media (CM) were collected for further purification and isolation of EVs, as described below.

### 2.6 Isolation of tEVs from the cell culture

Cell culture medium containing tEVs was pre‐cleared first by a low‐speed centrifugation step (700 × *g* for 5 min), followed by centrifugation at 2000 × *g* for 10 min to remove larger particles and debris. Unless indicated otherwise, samples were subsequently filtered through a bottle top filter (SARSTEDT AG & Co. KG, Germany) with a cellulose acetate membrane with a 0.22 µm pore size to remove any remaining larger particles. The CM was then ultra‐filtrated using tangential flow filtration (MicroKross, 20 cm^2^, SpectrumLabs) with a cut‐off of 300 kDa to concentrate the culture medium. The concentrated retentate was further concentrated with an Amicon Ultra‐15 10 kDa molecular weight cut‐off spin‐filter (Millipore) to a final volume of 100 µL, as previously described (Lennaárd et al., 2021). Size exclusion chromatography (SEC) was performed using qEV 70 nm columns (for EVs‐isolation; Izon Science), collecting fractions 4 and 5 in 2 mL total, then the fractions were concentrated using Amicon® Ultra‐15 Centrifugal Filter Units (10 kDa; Merck) and stored at −80°C for further analysis.

### 2.7 Nanoparticle tracking analysis

The size distribution and concentration of EVs purified from the B16F10 cell culture were determined employing the NS500 nanoparticle analyser (NanoSight, Malvern, Worcestershire, UK) as previously described (Lennaárd et al., 2021). In brief, diluted samples (100×–10,000×) were loaded into the NTA device, and five 30‐s videos were recorded for each sample with a 7‐s delay between recordings and a camera level of 14–15. The analysis was performed with the screen gate set at 10 and the detection threshold at 7, with all remaining settings set to automatic.

### 2.8 Cryo transmission electron microscopy

For sample preparation, a 2/2 mesh 300, Cu sample grid (Quantifoil, Germany) were glow‐discharged (20 mA, 60 s). Then 3 µL of the sample was applied. The grids were blotted for 8 s, at 100% humidity at 16°C with a Vitrobot (ThermoFisher, Netherlands) and vitrified into liquid ethane. The grids were imaged with a Talos Arctica at 200 kV (ThermoFisher, Netherlands). The images were collected on a Falcon III (ThermoFisher, Netherlands) camera in integrating mode and the images were summed without motion correction at a dose of 50 electrons/Å^2^ and a defocus of −3 µm at a nominal magnification of XX (The images were visualized with ImageJ.).

### 2.9 Characterization of markers on tEVs

Purified tEVs were diluted to 1 × 10^10^ particles/mL concentration with DPBS. Then, 2.5 × 10^8^ EVs in 25 µL were stained with and without 8 nM of PE conjugated antibody against CD63 (BD Biosciences, Stockholm, Sweden), a surface marker enriched in exosomes. In a sealed 96‐well plate (SARSTEDT AG & Co. KG, Germany), the EVs were incubated overnight at room temperature in the dark, followed by a subsequent dilution to produce 1 × 10^7^ EV/mL in a final volume of 100 µL and without antibody. Single‐vesicle imaging (Amnis Cellstream; Luminex) was performed to analyse the small particles. The results were analysed by dedicated software (FlowJo v. 10.8; FlowJo LLC).

### 2.10 Bicinchoninic acid (BCA) assay

Protein concentrations for tEVs and B16F10 cell lysates were measured by a micro‐BCA assay (Thermo Fisher, Cat No. 23235) following the manufacturer's instructions.

### 2.11 Western blotting for tEVs markers

The presence of the EV‐associated tetraspanins CD63 and CD9 and the intravesical protein Alix were determined using Western blot. Briefly, 2 × 10^10^ EVs (or 15 µg of tEV) and 2 × 10^6^ B16F10 cells (or 15 µg of tEV) were lysed in 100 µL of radioimmunoprecipitation buffer (RIPA; BioRad, Hercules, CA, USA). The samples were incubated on ice for 30 min and vortexed for 10 s every 5 min. Then the tEV and cell lysates were mixed with 8 µL of loading buffer (10% glycerol, 8% sodium dodecyl sulfate, 0.5 M dithiothreitol, and 0.4 M sodium carbonate), incubated at 70°C for 5 min before loading onto the NuPAGE+ (Invitrogen, Novex 412% Bis‐Tris gel), and run at 120 V for 1.5 h. The proteins were transferred using the iBlot system (iBlot 2 Dry Blotting System; Invitrogen) for 7 min to an iBlot membrane (iBlot 2 Transfer Stacks; Invitrogen). The membrane was incubated with blocking buffer (Odyssey Blocking Buffer; LI‐COR Biosciences, Lincoln, NE, USA) at room temperature for 1 h and subsequently incubated overnight at 4°C with newly prepared primary antibodies (anti‐CD9 (10626D, Invitrogen) diluted 1:200, anti‐CD63 (10628D, Invitrogen) diluted 1:200, anti‐Alix (sc‐53540, Santa Cruz) diluted 1:400), anti‐TSG101 (MA1‐23296, Invitrogen) diluted 1:500), anti‐B‐actin (MA1‐91399, Invitrogen) diluted 1:5000, and anti‐PDL1 (14‐5982‐82, Invitrogen) diluted 1:500. The membranes were washed four times with TBS‐T 0.1% for 5 min each on a shaker before being incubated for 60 min with a 1:10,000 diluted secondary antibody (goat anti‐mouse, C00322). PBS was used three times to wash the membrane, and the results were visualized by an infrared imaging system (LI‐COR Odyssey CLx).

### 2.12 Heat inactivation of tEVs

SEC‐purified EVs derived from B16F10 cells were subjected to heat inactivation. A concentration of 1 × 10^11^ EVs per 100 mL was utilized. The heat inactivation process involved using a heating block to incubate the EVs at either 100°C for 10 min (for in vivo studies) or 56°C (for in vitro studies). As a control group, 100 µL of PBS was similarly heated at 100°C for 10 min. In the case of in vivo studies, the heat inactivation procedure was carried out freshly before each in vivo treatment over a span of 5 consecutive days.

### 2.13 Mice

Female C57BL/6N (H‐2^b^) mice at 8–10 weeks of age were purchased from Charles River (Charles River Laboratories, Germany). The animals were kept in standard cages at 21°C–22°C with a 12‐h light/dark cycle and 50% humidity at our animal facility (Karolinska University Hospital, NOVUM). All mice were given ad libitum access to irradiated standard mouse chow and tap water and allowed to acclimatise for one week before inoculation with tumour cells. All experiments in this study were pre‐approved by the Stockholm Southern Ethics Committee for Animal Research and were conducted in accordance with the animal welfare law, the Animal Protection Regulation and the Regulation of the Swedish National Board for Laboratory Animals (approval number: 3566, 5.2.18‐16738/18).

### 2.14 Tumour induction

In this study, we used the subcutaneous melanoma mouse model as an animal model for malignant melanoma in human. For tumour induction, B16F10 cells at a confluency of ≤80% were first harvested from the culture flasks by trypsinization and centrifuged at 400 × *g* for 5 min. Thereafter, supernatants were collected, aliquoted and stored at −20°C for later use. The pellets containing B16F10 cells were washed in DPBS and adjusted to a concentration of 5 × 10^6^ cells/mL in DPBS prior to inoculation. For tumour induction, mice were injected subcutaneously (s.c.) into the right flank of mice with 5 × 10^5^ tumour cells (100 µL of the cell suspension). Tumour volumes were evaluated every 3rd day by measuring two perpendicular diameters with callipers. Tumour volume (*V*) was calculated using the following equation: *V* = (*W × W × L*)/2, where *W* is the width of the tumour (small diameter), and *L* is the length (large diameter), both in millimetres.

### 2.15 Collection of blood and body organs

Mice with advanced tumours (14–21 days after tumour cell inoculation), were weighed and thereafter, bled by cardiac puncture under isoflurane anaesthesia and thereafter euthanized by cervical dislocation. The spleens, livers, femur and tibia bones were dissected out. The livers were used for histological analysis, and the spleens were cut into two pieces; one was used for histological analysis, and the second for flow cytometry.

Blood samples were collected in blood collection tubes (Microtainer, BD Bioscience, NJ, USA) containing either EDTA dipotassium (for the analysis of haematological parameters), haemoglobin concentration was performed using the alkaline hematin method based on the manufacturer's instructions (51280‐1G, Sigma‐Aldrich) or no additive (for the analysis of enzymes and cytokines). Serum preparation and storage were performed as described previously (16).

### 2.16 Preparation of cell suspensions from the spleen and the bone marrow

Spleens were gently teased apart with forceps in PBS containing 1% FCS and washed and re‐suspended in the same buffer before use. Bone marrow cells were flushed out of the femur and tibia with PBS containing 1% FCs, using a syringe with a 27‐gauge needle. These cells were also washed and re‐suspended in the same buffer prior to use.

### 2.17 Histological analysis of the spleen, lymph nodes, and liver

Spleens and livers were fixed in 4% (v/v) formaldehyde in PBS for 24 h, placed in 70% (v/v) ethanol for 24 h, and then embedded in paraffin. Sections (5−6 µm) were stained with haematoxylin and eosin (H&E) for examination by light microscopy (ZEISS, Carl Ziess, AG, Germany). The images obtained were converted to an EGB colour format with the Adobe Photoshop CS4 program (Adobe systems, San Jose, CA, USA).

### 2.18 Measurement of arginase activity

The activity of arginase in serum samples or cell culture supernatants was quantified employing a commercially available kit (Merck/Sigma, Stockholm, Sweden) in accordance with the manufacturer's instruction.

### 2.19 Proteomic profiling of angiogenesis factors and cytokines/chemokines

The relative expression levels of 31 proteins associated with mouse angiogenesis and 40 cytokines and chemokine in 1 mL cell culture supernatants and/or 2 × 10^11^ tEVs (100 µL) derived from B16F10 cells were visualized using a proteome profiler mouse angiogenesis array kit (R&D Systems, Minneapolis, MN, USA). Prior to performing the experiment, the tEVs were lysed by Mammalian Cell Lysis Buffer 5× (ab17983). Then the assay was performed in accordance with the manufacturer's instruction, except for the last step in which Tetramethylbenzidine (TMB) liquid substrate system (Sigma‐Merck, Darmstadt, Germany) was used for the colour development. Pixel colour densities on the membranes were analysed using the image analysis software, ImageJ.

### 2.20 Proteinase K treatment

About 5 × 10^11^ tEVs were incubated with 100 µg/mL Proteinase K (Qiagen) at 37°C for 3 h prior to angiogenesis factors and cytokines/chemokines measurement. The tEVs were purified by SEC using qEV 70 nm columns (for EV isolation; Izon Science) as described above. Then 2 × 10^11^ were used for measurement of angiogenesis factors, and 2 × 10^11^ were used for measurement of cytokines and chemokines as described above.

### 2.21 Immunofluorescent staining and flow cytometric analysis

Immunophenotyping of bone marrow, spleen cells and bone marrow cell‐cultured cells was performed using a panel of antibodies against the appropriated murine cell surface antigens. The antibodies employed were the following: anti‐mouse Gr1 antibody labelled with (−) R‐phycoerythrin (PE, clone RB6‐8C5), rat anti‐mouse CD11b antibody labelled with (−) allophycocyanin (APC, clone M1/70), rat anti‐mouse CD19 labelled PE (clone 1D3), rat anti‐mouse CD9 labelled APC (clone KMC8), rat anti‐mouse CD71 labelled PE (clone C2) and rat anti‐mouse TER 119 labelled APC (clone TER 119) (all antibodies were from Pharmingen, BD Biosciences, Stockholm, Sweden).  Organ or cell culture derived cells (10^6^ cells) suspended in 50 µL DPBS were added to each individual well of 96‐well round‐bottom tissue culture plates (Corning, NY) and thereafter, incubated with antibodies in the dark and on ice for 30 min, in accordance with the recommendations of the manufacturer (BD Pharmingen; San Diego, CA). Subsequently, the cells were washed and re‐suspended in 400 µL DPBS. The stained cells were further analysed utilizing FACSArray flow cytometer (Becton Dickinson, San Jose, CA). For each sample, the data from 10,000 events (individual cells) were collected and analysed using the CellQuest Software.

### 2.22 In vivo treatment with tEVs derived from B16F10 melanoma cells

To relatively mimic the condition for the production of tEVs at the immune escape phase, a group of female C57BL/six mice (five mice/group) were injected intraperitoneally (i.p.) with tEVs (with or without heat inactivation) at a concentration of 1 × 10^11^ particles/mouse for 5 subsequent days. An untreated group of the same strain was injected with PBS and used as the control group. On day 8, the injected animals were weighed, bled by cardiac puncture under isoflurane anaesthesia, and thereafter euthanized by cervical dislocation. The spleen and the femur and tibia bones were dissected out, and thereafter, haematological, histological, biochemical, and flow cytometric analyses of the blood and organs were performed as described above.

### 2.23 Ex‐vivo evaluation of tEVs derived from B16F10 melanoma cells on bone marrow hematopoietic cells

To investigate the potential direct effect of tEVs on hematopoietic cells, bone marrow cells were isolated from healthy mice and seeded in a 24‐well format at a concentration of 75 × 10^5^ cells in 500 µL per well, using RPMI supplemented with 20% FBS, 1% penicillin‐streptomycin (5000 U/mL, Gibco™ Life Technology), and 50 µM 2‐mercaptoethanol (Sigma Aldrich). While cells were seeded, the tEVs were added to the wells as follow: 5 × 10^9^ non‐heated tEVs, 5 × 10^9^ 56°C heated tEVs, and 5 × 10^9^ 100°C heated tEVs. Three days after incubation in a humidified incubator at 37°C and 5% CO_2_, cells were stained with Gr1/CD11b and CD71/TER119 and analysed by flow cytometry with a MACSQuant Analyzer 10 flow cytometer (Miltenyi Biotec). FlowJo (v. 10.8.1, FlowJo) was used to analyse flow cytometry data.

### 2.24 Live cell imaging assays

Live cell imaging was performed as previously described [reference]. The IncuCyte machine was placed in a humidified incubator at 37°C and 5% CO_2_. 75 × 10^5^ bone marrow cells were seeded per well in a 24‐well plate format as described above. While cells were seeded, the tEVs were added to the wells as follows: 5 × 10^9^ non‐heated tEVs, 5 × 10^9^ 56°C heated tEVs, and 5 × 10^9^ 100°C heated tEVs. The confluency of the cells was monitored (phase contrast, nine images per well) every day for 5 days using an IncuCyte® S3 Live Cell Analysis System (Sartorius).

### 2.25 Cell uptake experiment

Bone marrow cells from healthy mice were collected and cultured in a 46‐well plate with 1 × 105 cells in 250 µL of RPMI that had 20% FBS, 1% penicillin‐streptomycin (5000 U/mL, GibcoTM Life Technology), and 50 µM 2‐mercaptoethanol (Sigma Aldrich). While cells were cultured, the CD63‐mNG‐tEVs were added to the wells as follows: 5 × 10^9^ non‐heated CD63‐mNG‐tEVs and 5 × 10^9^ 100°C heated CD63‐mNG‐tEVs. After 4 h of incubation in a humidified incubator at 37°C and 5% CO_2_, cell uptake was analysed by flow cytometry with a MACSQuant Analyzer 10 flow cytometer (Miltenyi Biotec). FlowJo (v. 10.8.1, FlowJo) was used to analyse flow cytometry data.

### 2.26 Ex vivo inhibition assays for angiogenic factors

The inhibitory effect of pazopanib, a VEGFR inhibitor (SML3076, Sigma‐Aldrich, Germany), pioglitazone, an osteopontin inhibitor (E6910, Sigma‐Aldrich, Germany), epigallocatechin, an ADAMTS‐1 inhibitor (E3768, Sigma‐Aldrich, Germany), and rmTFPI Protein, a recombinant mouse tissue factor pathway inhibitor (2975‐PI, R&D Systems) were evaluated to assess their abilities to prevent the possible impact of tEVs on the hematopoietic. Bone marrow cells were extracted from healthy mice and thereafter, placed in a 24‐well format at a concentration of 75 × 105 cells in 500 µL per well or 1.5 × 10^5^ cells in 250 µL per well following the method outlined earlier. Subsequently, the cultured cells were exposed to the above‐mentioned inhibitors of angiogenic factors at different concentrations (e.g., pazanopanib (10 and 20 µg), pioglitazone (50, 100, 200, 400 µM), epigallocatechin (10 nM, 100 nM, 1000 nM, 10 µM) and rmTFPI (10 nM, 100 nM, 1000 nM, 10 µM)) for a duration of 2 h after which the cells were treated with 5 × 10^9^ tEVs or 2.5 × 10^9^. Thereafter, the cultured cells were incubated in a controlled environment set at a temperature of 37°C and a CO_2_ concentration of 5% for 3 days, after which, the cell proliferation was assessed using the WST‐1 method outlined below.

### 2.27 Cell proliferation assay

For the cell proliferation assay, WST‐1 was employed. Bone marrow cells were isolated from healthy mice and seeded in a 24‐well format at a concentration of 75 × 10^5^ cells in 500 µL per well and treated with or without pazopanib and tEVs as described above. After 72 h, 10 µL of WST‐1 were added to each well for 4 h at 37°C and 5% CO_2_ according to the manufacturing protocol. Then 100 µL of the media were transferred to a 96‐well plate. Then the plate was read at 450–650 nm using a fluorometer (SpectraMax® i3x; Molecular Devices LLC, San Jose, CA, USA).

### 2.28 Statistical analysis

Data are presented as means ± standard deviation (SD). Depending on the experiment, statistical analysis was performed using either a two‐tailed Student's *t*‐test or the Mann–Whitney test (*U*‐test) or using one way analysis of variance (ANOVA) with each value Tukey's multiple comparisons test. A *p*‐value ≤ 0.05 was considered statistically significant. All statistical analyses were performed with GraphPad Prism9 software.

## 3 RESULTS

### 3.1 Characterization of tEVs derived from B16F10 melanoma cells

**FIGURE 1:**
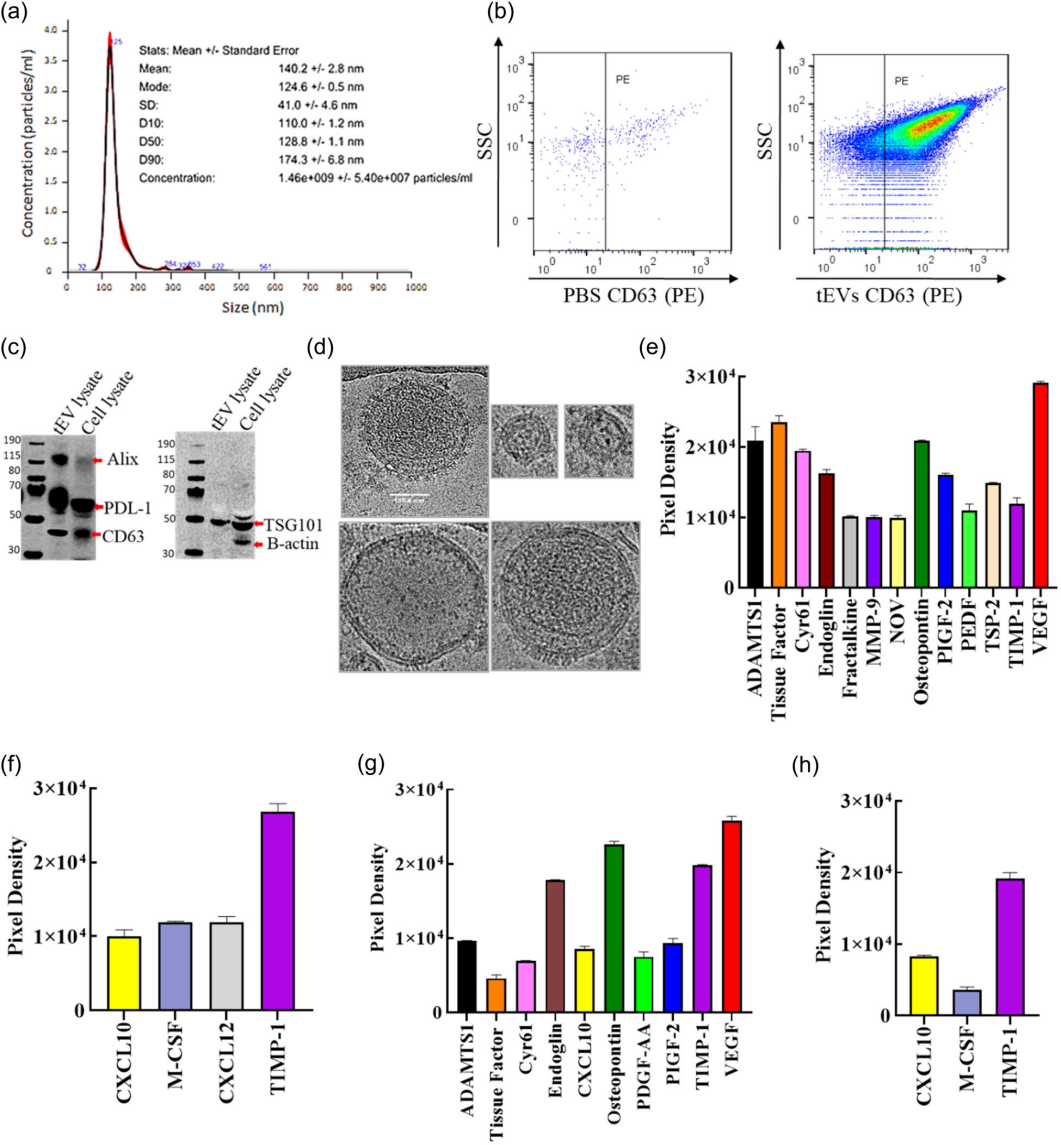
Characterization of tEVs isolated from B16F10 cells and proteomic profiling. (a) Nanoparticle analysis of tEVs demonstrates single‐peak distribution within a 100–200 nm range consistent with functional EV subsets such as exosomes and microvesicles. (b) Using high‐resolution imaging flow cytometry (Amnis CellStream) and tetraspanin‐labelled phycoerythrin (PE) anti‐mouse, the EVs marker CD63 was identified in accordance with the forward scatter signal (FCS). (c) Western Blot analysis shows that tEVs are positive for the common melanoma‐tEV markers CD63, Alix, PDL‐1, and TSG101 and negative for B‐actin. (d) cryoEM imaging confirms tEV particles within the range of the nanoparticle analysis (scale bar 100 nm). (e) Histogram profiles for the differential expression of 13 angiogenic factors presence in/on tEVs was screened using Proteome Profiler Arrays, calculating the mean spot pixel densities with standard imaging software. (f) Histogram profiles for the differential expression of four cytokines presence in/on tEVs was screened using Proteome Profiler Arrays, calculating the mean spot pixel densities with standard imaging software. (g) Histogram profiles for the differential expression of 10 angiogenic factors presence in 1 mL of B16F10 supernatant for 3 days, was screened using Proteome Profiler Arrays, calculating the mean spot pixel densities with standard imaging software. (h) Histogram profiles for the differential expression of four cytokines presence in 1 mL of B16F10 supernatant for 3 days, was screened using Proteome Profiler Arrays, calculating the mean spot pixel densities with standard imaging software. The data are shown as means (±SD, *n* = 2). (e–h) The experiment has been repeated similarly twice.

To explore the possible involvement of tEVs in the development of immune escape phase of cancer immunoediting and tumour‐induce dysregulated haematopoiesis, the tEVs from cultured B16F10 melanoma cells were purified by SEC and characterized. The isolated tEVs had a mode size of 125 nm as demonstrated by nanoparticle tracking analysis (NTA) (Figure 1a). Flow cytometric analysis revealed that more than 90% of the detected tEVs expressed high levels of the EV marker CD63 (Figure 1b), which was also detected on the cells (Fig. S1). Furthermore, Western blot analysis showed that EVs derived from B16F10 cells also contained ALG‐2‐interacting protein X (Alix) as well as the tetraspanins CD9, CD63, PDL‐1, TSG101 and B‐actin (Figure 1c, Figure S1c and d). In addition, morphological characterization by cryo‐EM revealed that the prepared EVs contained large and small variants of vesicles that were mainly intact and had a round shape. There is a visible density at the lipid bilayer membrane as well as evidence for electron‐dense cargo (Figure 1d). We next sought to identify their protein cargo contents. To this end, proteomic analysis of angiogenesis factors and chemokines in the isolated EVs from B16F10 melanoma cells was performed. As shown in Figure 1e and Figure S2c, B16F10 cell‐derived EVs contained elevated levels of different angiogenic factors in accordance with the following decreasing level values: VEGF > tissue factor > osteopontin ≥ ADAMTS1 > Cyr61 > endoglin = PIGF‐2 > TSP‐2 > TIMP‐1 > PDGF > fractalkine = MMP‐9 = NOV. This observation strongly implied that B16F10 EVs were highly enriched in different growth and angiogenic factors. Next, to investigate whether chemokines were encapsulated into or bound to tEVs, cytokine array was performed, which showed that four significant expression levels of the chemokine TIMP‐1 > CXCL12 = M‐CSF > CXCL10 (Figure 1f and Figure S2f). This finding strongly suggested that EVs produced from B16F10 cells were considerably enriched in several chemokines. To ascertain whether these factors resided within or on the surface of EVs, we conducted proteinase K treatments. This revealed that certain angiogenesis factors, such as Cyr61, Endoglin, Fractalkine, MMP9, PIGF‐2 and PEDF, disappeared, as depicted in Figure S2i and j. Similarly, cytokines like CXCL10 and M‐CSF vanished, as illustrated in Figure S2g and h. However, other angiogenesis factors and cytokines were unmasked, as shown in Figure S2 g–j. Additionally, we observed that the particle size reduced when treated with proteinase K compared to untreated samples, measuring 95 and 125 nm, respectively (Figure S2k, Figure 1a).

### 3.2 Identification of secreted factors from B16F10 melanoma cells

Following the finding that EVs derived from B16F10 cells contained several angiogenic factors, we next asked whether these factors were also released as soluble factors by B16F10 cells. Thus, proteomic profiling of angiogenic factors and cytokines was performed on the conditioned culture medium of B16F10 melanoma cells. As shown in Figure 1g, Figure S2b, comparable to EVs derived from B16F10 cells, this culture medium contained substantial amounts of VEGF, osteopontin, TIMP‐1, endoglin, PIGF‐2, ADAMTS1, cyr61, PDGF and tissue factor. However, in contrast to B16F10 cells derived EVs, the cell culture medium exhibited significant expression level of only three chemokines TIMP‐1 > CXCL10 along with low expression levels of M‐CSF (Figure 1h, Figure S2e). We further investigated to see whether these factors were released by primary fibroblast cells (Figure S2i) and saw that no cytokines were made (Figure S2m) in the conditioned media. However, some angiogenesis factors were shown (Figure S2n and o) in conditioned media.

### 3.3 Advanced stage of melanoma coincides with a dysregulated haematopoiesis along with the expansion of immunosuppressive erythroid progenitor cells and MDSCs

**FIGURE 2:**
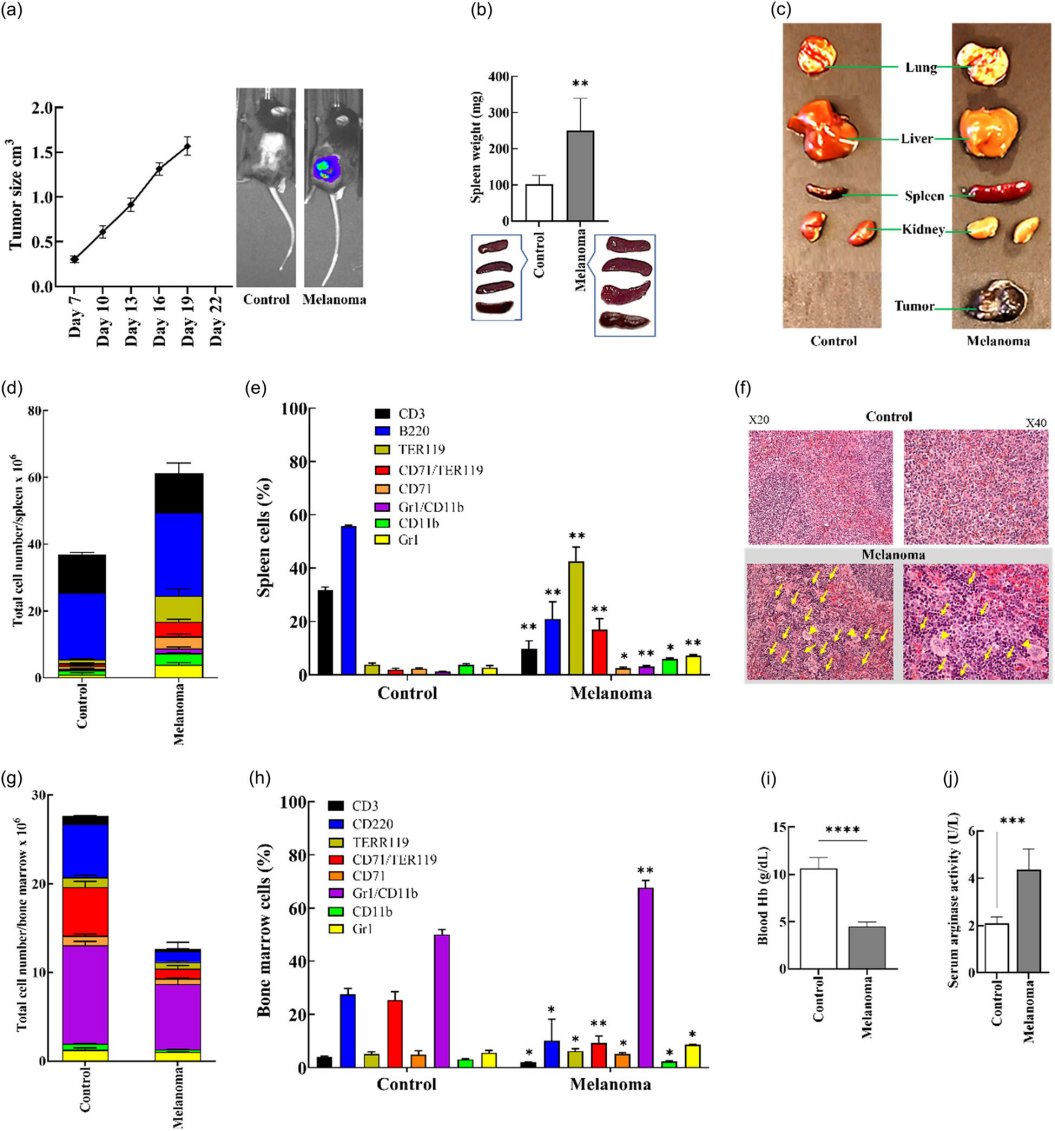
Tumour development and necroscopic features of mice with advanced stage B16F10 tumours. (a) Female C57BL/6N mice were either left un‐injected (control, *n* = 4) or subcutaneously injected with 5 × 10^5^ B16f10 cells (*n* = 4), and tumour sizes were tracked until they reached the advanced stage. (b) The spleens were excised and weighed at the end of the experiment. (c) Organ differences between tumour‐bearing and non‐bearing mice show the enlargement of spleen and anaemia in tumour‐bearing mice. The advanced stage of tumour development is characterized by the emergence of immunosuppressive MDSCs and EPCs, increased extramedullary haematopoiesis in the spleen and aberrant myelopoiesis in the bone marrow. Flow cytometry was employed to assess both the total count of distinct cell types (calculated as the total spleen cell numbers multiplied by the frequency of distinct cell type) (d) and the frequency of these distinct cell types within the spleens (e). (f) Representative microscopic (H&E staining) images (magnification 20× and 40×) of a tumour‐free mouse spleen exhibiting normal histological structure (top panel), or with the red pulp comprising erythropoietic foci (yellow arrowhead) and megakaryocytes (bottom panel). Flow cytometry was employed to assess both the total count of distinct cell types (calculated as the total bone marrow cell numbers multiplied by the frequency of distinct cell type) (g) and frequencies panel (h) of distinct bone marrow cell types. (i) Haemoglobin concentration using the alkaline hematin method. (j) Serum level of arginase was determined using either colorimetric assays or ELISA method. The data are presented as means (±SD, *n* = 3). Differences between the groups were analysed statistically employing Mann–Whitney *U* test (*p < 0.05, **p < 0.01, ***p ≤ 0.001).

Having observed that both EVs and conditioned medium from B16F10 cells shared substantial similarities in expressing growth factors and cytokines prompted us to evaluate whether EVs derived from the tumour cells can also contribute to tumour‐induced dysregulation of haematopoiesis. In this respect, we first performed an in vivo study to characterize the status of dysregulated haematopoiesis in mice bearing B16F10 melanoma. Subcutaneous inoculation of B16F10 tumour cells into C57BL/6 mice resulted in the development of advanced stage melanoma tumours (approximately 1.5 cm^3^) within 20 days (Figure 2a). Furthermore, at necropsy, in comparison to the tumour‐free control animals, tumour bearing mice exhibited splenomegaly (Figure 2b), as well as a pale appearance of the liver, kidney and lung (Figure 2c).

**FIGURE 3:**
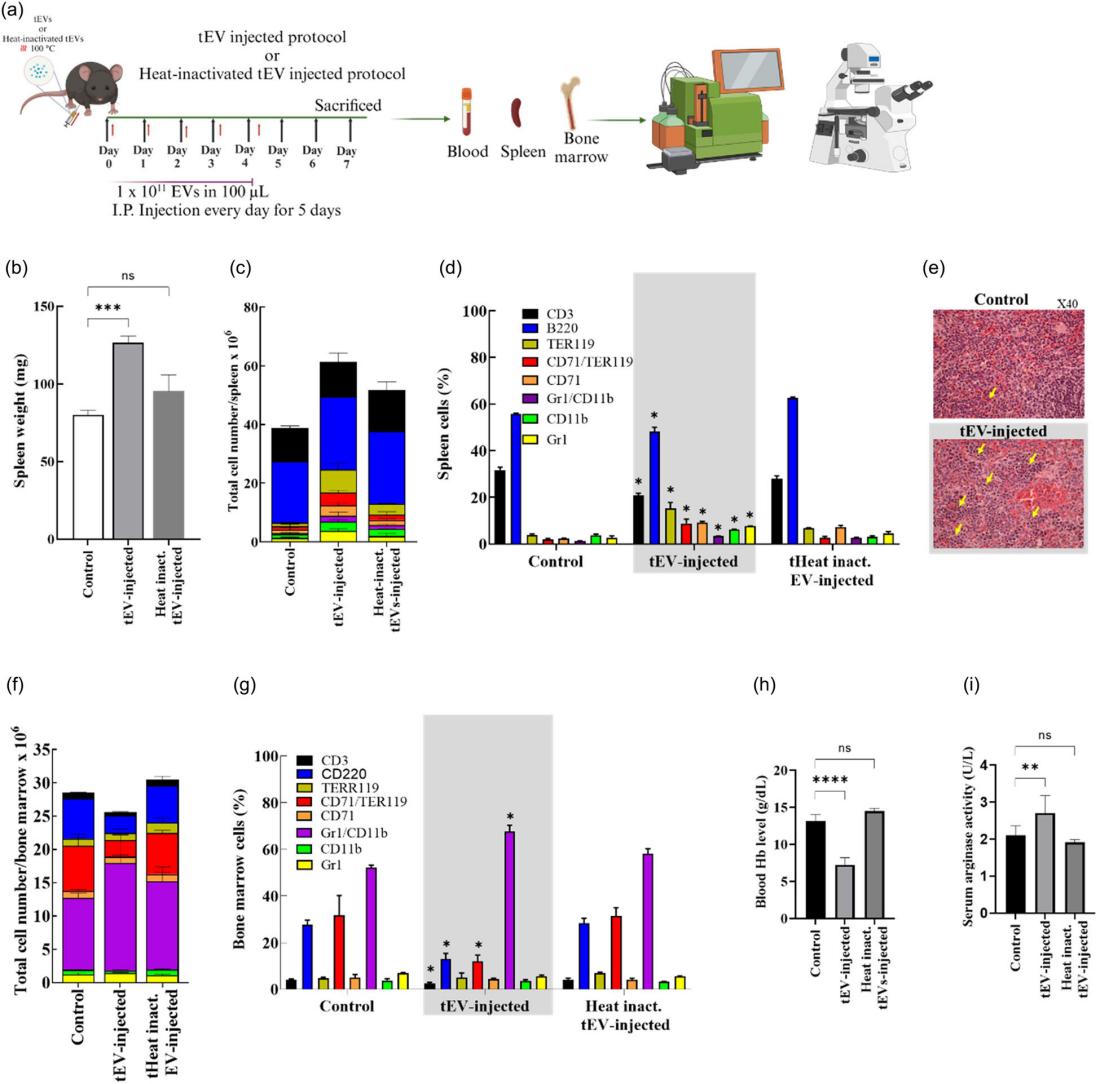
Induction of immunosuppressive EPCs and MDSCs, increased extramedullary haematopoiesis in the spleen and the development of aberrant myelopoiesis in the bone marrow, by tEVs and heat‐treated tEVs. (a) Schematic illustration of tEVs workflow in vivo using female C57BL/6N mice that were either un‐injected (control, *n* = 3) or i.p. injected with 1 × 10^11^ tEVs or heat‐treated tEVs at 100°C every day for 5 days (*n* = 3). (b) The spleens were excised and weighed at the end of the experiment. Flow cytometry was employed to assess both the total count of distinct cell types (calculated as the total spleen cell numbers multiplied by the frequency of distinct cell types) (panel c) and frequencies (panel d) of distinct cell types in the spleens. (e) Representative microscopic (H&E staining) pictures (magnification 20× and 40×) of C57BL/6N mice with or without i.p. injected tEVs, spleen exhibiting normal histological structure (top panel), or red pulp comprising erythropoietic foci (yellow arrowhead) and megakaryocytes (bottom panel). (f and g) Flow cytometry was employed to assess both the total count of distinct cell types (calculated as the total bone marrow cell numbers multiplied by the frequency of distinct cell types) (panel g) and frequencies (panel h) of distinct bone marrow cell types. (h) Haemoglobin concentration using the alkaline hematin method K. (i) The serum level of arginase was determined using either colorimetric assays or the ELISA method. The data are presented as means (±SD, *n* = 3). Differences between the groups were analysed statistically using the Mann–Whitney *U* test (**p < 0.01, ***p < 0.001, ****p ≤ 0.0001).

The development of splenomegaly led us to characterize the status of immune cell populations using flow cytometry. As expected and shown previously (Kamran et al., 2018), these mice exhibited substantial elevations in the total numbers and frequencies of splenic EPCs (including both CD71+/TER119+ and TER119+ subpopulations) (Figure S3) in combination with a slight but significant increase in the frequencies of MDSCs (CD11+Gr1+) macrophages (CD11b+) and neutrophils (Gr1+) (Figure S4), as compared to those of their corresponding tumour free control mice (Figure 2d and e). Although melanoma bearing mice exhibited substantial reduction in the frequency of T (CD3+) and B (B220+) cells (Figure 2e, Figure S5), the absolute numbers of these cell populations were comparable in both groups (Figure 2d). These observations suggested the occurrence of extramedullary haematopoiesis (EMH)/erythropoiesis during the late stage of B16F10 melanoma. To further investigate this, histopathological analysis on the spleen was performed using standard H&E staining. As shown in Figure 3f, in comparison to tumour free animals (Figure 2f, upper panel), mice bearing B16F10 tumours showed an extensive EMH in the splenic red pulp characterized by the presence of clusters (foci) of erythroid precursor cells and scattered megakaryocytes (Figure 2f, lower panel).

It is well established that extramedullary erythropoiesis may emerge during cancer development, in order to maintain erythroid haemostasis while medullary erythropoiesis is suppressed (Grzywa et al., 2021). Thus, we next performed flow cytometry analysis on the bone marrow cells. As shown in Figure 2g, tumour bearing mice exhibited a substantial reduction in the total numbers of intermediate progenitors of erythrocytes (CD71+/TER119+), B cells, T cells, MDSCs (CD11+/Gr1+), and granulocytes (Gr1+). However, the same animals had increased frequencies of MDSCs (CD11+/Gr1+) and granulocytes (Gr1+) along with substantially decreased frequencies of B (CD19+) and T (CD3+) cells (Figure 2h, Figures S4 and S5). Furthermore, as a reflection of profound alterations in the splenic and bone marrow erythropoietic cells, tumour bearing mice displayed a severe anaemic status, that is, their blood haemoglobin values were substantially lower than those of the controls (Figure 2i). Finally, since it is well established that tumour‐induced expanded splenic EPCs and MDSCs induce immunosuppression via the production of several immunosuppressive factors, in particular arginase (Grzywa et al., 2021; Mittal et al., 2014), the circulatory levels of this enzyme were measured using a colorimetric method. As shown in Figure 2j, and in line with the expansion of EPCs, melanoma bearing mice exhibited significantly higher activity of arginase in their sera as compared to the sera of the corresponding tumour‐free controls.

### 3.4 tEVs induce extramedullary expansions of erythroid progenitors (EPCs) and MDSCs along with anaemia in mice

We next characterized tEVs to investigate their potential contribution to the immune escape phase of cancer immunoediting. First, we performed the experiment by injecting a low dose (1 × 10^10^) of tEVs i.p. into syngeneic mice for five consecutive days. We found that 1 × 10^10^ showed no effect on the expansion of MDSCs and EPCs in both the spleen and bone marrow (Figures S6 and S7). Then ten times more (1 × 10^11^) of tEVs were injected i.p. into syngeneic mice for five consecutive days (Figure 3a). After a resting period, we analysed the spleens, bone marrows, and blood samples for dysregulated haematopoiesis, anaemia and the presence of immunomodulatory factors. Our results showed that mice injected with tEVs exhibited splenomegaly (Figure 3b), increased total numbers and frequencies of erythroid (CD71+/TER119+ and TER119+ cells (EPCs and RBCs, respectively) (Figure S3) and myeloid cell populations (CD11b+/Gr1+, CD11b+ and Gr1+ cells (MDSCs, macrophages and neutrophils, respectively) (Figure S4). Similar to melanoma‐bearing mice, however, reduced frequencies, but not absolute numbers, of T‐ and B cells (CD3+ and B220+ cells, respectively) (Figure 3c and d, Figure S5). The tEV‐injected mice also showed increased erythropoietic foci in the spleen (Figure 3e). Furthermore, the immunophenotypic patterns of bone marrow cells in tEV‐injected mice resembled those of mice with melanoma, with increased total and frequency MDSCs (CD11b+/Gr1+ cells) and decreased total and frequencies of EPCs (CD71+/TER119+ cells), B cells (B220+ cells), and T cells (CD3+ cells) (Figure 3f and g, Figures S3–S5). These changes were accompanied by reduced blood levels of haemoglobin (Hb) (Figure 3h) and enhanced circulatory activity of arginase (Figure 3i). These observations collectively indicate that tEVs can induce dysregulated haematopoiesis with an immunoregulatory effect, similar to that observed in mice with tumours.

### 3.5 Induction of dysregulated haematopoiesis by tEVs is heat‐sensitive

Upon discovering that tEVs have the potential to disrupt haematopoiesis, we aimed to identify the specific determinants or components on tEVs responsible for inducing this dysregulation. To accomplish this, we considered that tEVs are integral parts of living cells and are composed of various macromolecules, including proteins, lipids, DNA, and RNA (Gurung et al., 2021; Rädler et al., 2023; Xu et al., 2018) (also indicated in this study). Additionally, we took into account that these components are sensitive to thermal denaturation, with lipid phase transitions occurring below 37°C, protein denaturation occurring between 40°C and 80°C, and DNA and RNA unfolding happening above 85°C–90°C (Bischof & He, 2005; Lepock, 1982, 2003). Consequently, we exposed tEVs to a temperature of 100°C for 10 min to effectively denature the bioactive molecules within these organelles before each i.p. injection (Figure 3a). As shown in Figure 3b–g, heating the EVs abolished the development of splenomegaly (Figure 3b), alterations in the splenic and bone marrow cell populations (Figure 3c and d), occurrence of anaemic status (Figure 3h) and enhancement of circulatory arginase activity (Figure 3i). However, heat‐inactivated tEVs‐injected animals exhibited a slight but significant increase in the numbers of B (CD19+) cells in the spleen and bone marrow (Figure 3c–g).

### 3.6 tEVs promote the expansion of MDSCs in cultured bone marrow cells ex‐vivo, a phenomenon that is inhibited by the blockade of VEGF

**FIGURE 4:**
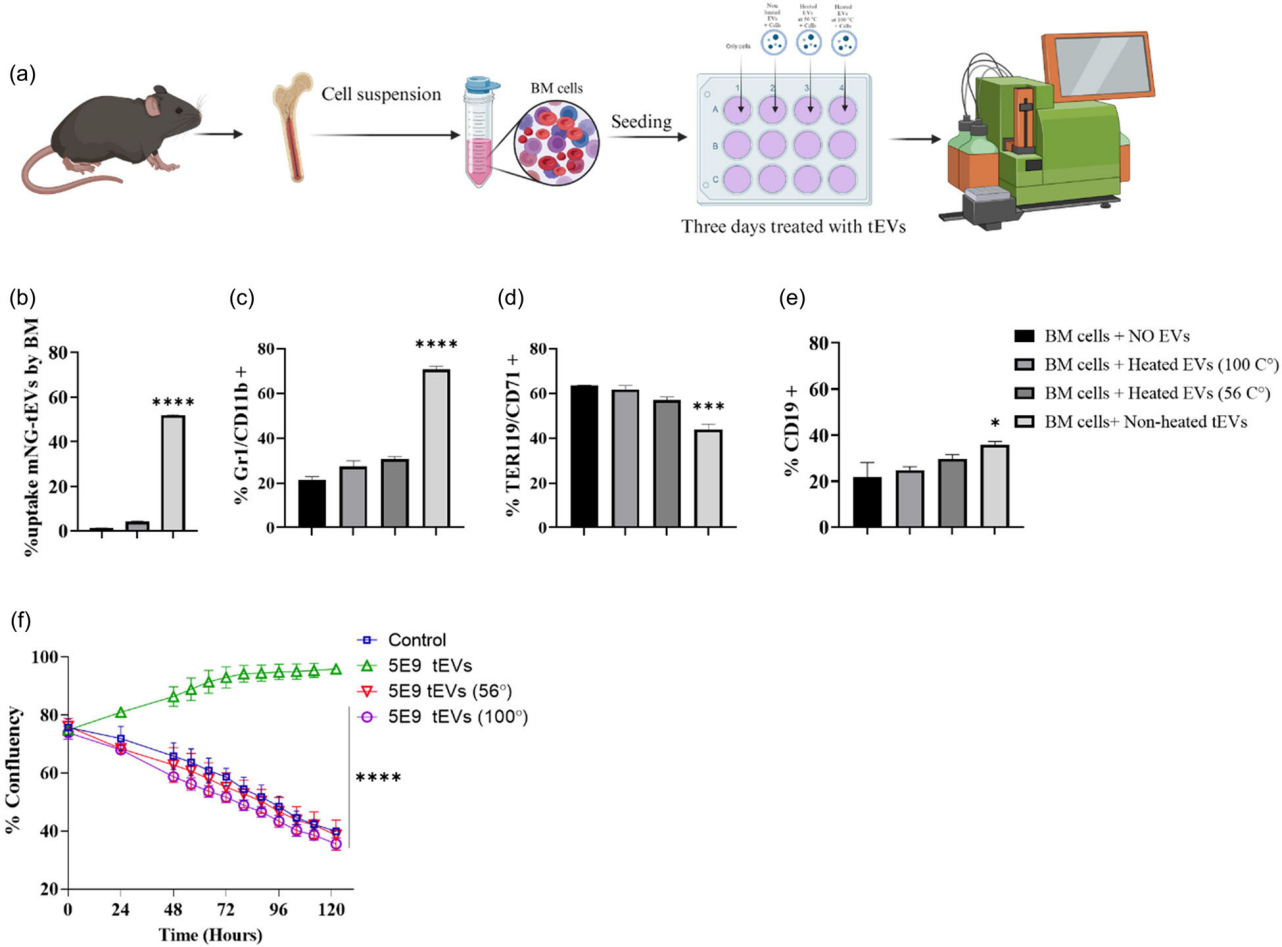
Effect of tEVs on bone marrow cells from female C57BL/6N mice. (a) Ex‐vivo experiment schematic illustration. (b) Internalization of fluorescent tEVs by BM cells after 4 h of incubation. (c–e) Flow cytometry was used to investigate the frequency of MDSCs (Gr1+/CD11b+), Late EPCs (CD71+/TER119+) and B cells (CD19+) in the bone marrow of female C57BL/6N mice with or without the addition of 5 × 10^9^ tEVs or tEVs heated at 100^o^C or 56^o^C. (f) IncuCyte S3 live‐cell analysis for assessing the activity of tEVs on BM cells. Data analysis was performed on real live‐cell images. Each dot represents the meaning of triplicate data. The data are presented as means (±SD, *n* = 3). Statistical significance (*p < 0.01, ***p < 0.001, and ****p < 0.0001) between experimental groups was calculated with using Mann–Whitney *U* test or one‐way analysis of variance (ANOVA) with each value Tukey's multiple comparisons test.

**FIGURE 5:**
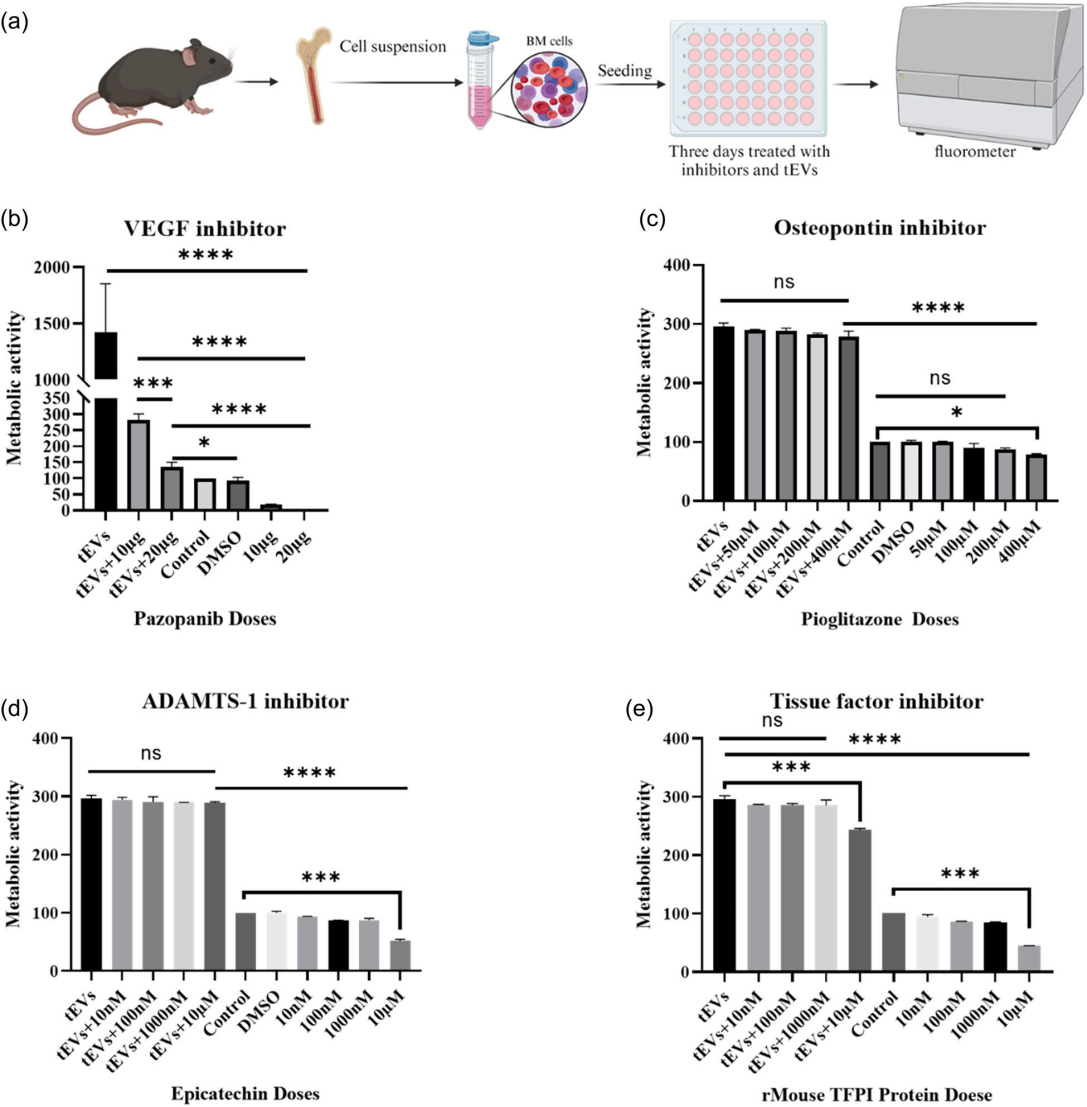
Effect of angiogenesis inhibitors on bone marrow cells from female C57BL/6N mice. (a) Schematic illustration of ex‐vivo experimental design. (b) Effect of pazopanib (VEGF inhibitor) on the inhibition of tEVs‐induced bone marrow cell proliferation. (c) Effect of pioglitazone (osteopontin inhibitor) on the inhibition of tEVs‐induced bone marrow cell proliferation. (d) Effect of epicatechin (ADAMTS‐1 inhibitor) on the inhibition of tEVs‐induced bone marrow cell proliferation. (e) Effect of rmTFPI (tissue factor pathway inhibitor) on the inhibition tEVs‐induced bone marrow cell proliferation. The cells were treated either inhibitors alone or with inhibitors in combination with 5e9 tEVs. The data are presented as means (±SD, *n* = 3). Statistical significance (*p < 0.01, ***p < 0.001, and ****p < 0.0001) between experimental groups was calculated with using one‐way analysis of variance (ANOVA) with each value Tukey's multiple comparisons test.

To determine whether tEVs derived from melanoma cells can directly trigger the expansion of MDSCs from their progenitors, syngeneic BM cells were directly exposed to the tEVs (with three different conditions: non‐heated, heated at 56°C and/or heated at 100°C) without any other additional additives for 72 h (Figure 4a). First, the uptake experiment for fluorescent tEVs was performed. It was shown that fluorescent tEVs were internalized by BM cells after 4 h of incubation (Figure 4b, Figure S8). Thereafter, the frequencies of cell populations belonging to the myeloid and erythroid lineages were determined. As shown in Figure 4c–e, and Figure S9, non‐heated tEVs exposure of BM cells induced the expansion of MDSCs (CD11b+Gr1+ cells) and B cells (CD19+) while simultaneously inhibiting the frequency of EPCs. It was hypothesized that the administration of higher doses of tEVs would result in an augmented proliferation of MDSCs and B cells while concurrently reducing the frequency of EPCs in BM cells. To confirm this, we administered two different doses (5e9 and 1e10) of tEVs. It was shown that both 5e9 and 1e10 tEVs had similar effects on expansion of MDSCs and B cells and reducing the frequency of EPCs in BM cells (Figures S10 and S11). For further confirmation of the biological role of tEVs in BM cells, we performed a live imaging experiment for the live cell proliferation assays. tEVs were added to the BM cells at three different conditions: non‐heated, heated at 56°C, heated at 100°C, or without any other additional additives for 5 days. It was observed that the non‐heated tEVs induced cell proliferation resulting in increased cell confluence from 24 to 72 h, and reached the plateau level after 80 h. However, the confluency of the cells started to decline at 24 h in other conditions (Figure 4f, Figure S12a). This finding indicated the possibility that functional effector molecules inside or on the surface of tEVs govern the expansions of BM‐derived MDSCs and B cells. To evaluate this indication, we selected VEGF as the most potent and increased angiogenic factor present on the tEVs (Figure 1f) and is known to function as a pivotal factor in haematopoiesis (Podar & Anderson, 2005). Hence, we used pazopanib, a vascular endothelial growth factor receptor (VEGFR) inhibitor (Podar et al., 2006) to elucidate the role of VEGF in the tEV‐induced expansion of MDSCs and B cells. As shown in Figure 5b, Figure S12b, pazopanib significantly inhibited the tEV‐induced BM cell expansion in a dose‐dependent manner.

In addition to VEGF, tEVs also contained high levels of other angiogenic factor including tissue factor, ADAMTS‐1, and osteopontin (OPN) (Figure 1f). To evaluate whether any of these factors plays a role in the expansion of BM cells induced by tEVs, we used rmTFPI Protein, epigallocatechin and pioglitazone, interfere with activation pathway induced by tissue factor, ADAMTS‐1, and osteopontin, respectively. As shown in Figures 5c–e, in the absence of tEVs, either of these compounds inhibited the expansion of BM cell expansion only at the highest used doses. On the other hand, in the presence of tEVs, while epigallocatechin and pioglitazone were ineffective, rmTFPI was able to slightly but significantly inhibit the tEV‐induced BM cell expansion at the highest used dose.

## 4 DISCUSSION

In this study, we conducted experiments with the aim of obtaining a thorough understanding of the role played by EVs derived from cancer cells in the developing the immune escape phase of cancer immunoediting. Our particular focus was investigating how these tEVs contribute to the disruption of haematopoiesis, a phenomenon known for its various immunosuppressive properties (Tao & Guo, 2020). As the model system, we used B16F10 melanoma cells which has been generated as the 10th serial passage subclone of the B16 parent tumour line in C57BL/6 mice and is commonly used to model aggressive metastatic melanoma in preclinical studies (Nakamura et al., 2002). Our initial set of findings indicated that EVs purified from B16F10 cells had diameters ranging from 100 to 200 nm. These tEVs exhibited expressions of CD63, CD9, and Alix, and possessed a spherical shape with a protein‐decorated lipid bilayer membrane. These physical, chemical, and morphological characteristics align closely with those typically observed in small EVs, including exosomes and microvesicles (Ratajczak & Ratajczak, 2020). Hence, like tumour cell derived soluble factors, tEVs can serve as mediators of extracellular communication during the development of tumour progression and immune escape phase. Supporting this notion, our protein profiling analysis demonstrated that both the cell culture media and EVs obtained from B16F10 melanoma cells displayed a comparable profile of angiogenic factors. Notably, VEGF, osteopontin, tissue factor, ADAMTS1, endoglin, PIGF‐2, TSP‐2, TIMP‐1, and PDGF emerged as the most abundant factors in both sample types. Indeed, most of these factors, particularly, VEGF, osteopontin, PIGF, and PDGF have also been found in patients with melanoma (Mahabeleshwar & Byzova, 2007; Peris‐Torres et al., 2020; Zhao & Huang, 2021). Thus, it is conceivable that during the immune escape phase, the aberrant release of these angiogenic factors, either in soluble form or integrated into EVs, by tumour cells can have severe adverse effects on the cellular components and functions of distant organs. In line with this, here, mice harbouring advanced melanoma exhibited splenomegaly, as well as a notable pallor in the liver, kidney, and lungs. Moreover, studies have demonstrated that in mice with B16F10 melanoma, the liver pallor is linked to metabolic dysfunction in this organ and the onset of fatty liver disease (Wang et al., 2023). The findings of this study establish a connection between the development of splenomegaly and the emergence of tumour‐induced EMH which is characterized by the expansion of MDSCs and EPCs. Furthermore, and of significant importance, the reduced cellularity and impaired erythropoiesis process in the BM can also be attributed to the abnormal release of angiogenic factors derived from B16F10 melanoma cells. Given the ability of cancer cells to release EVs that can systematically impact the function of both local and distant organs, our study demonstrated that administration of EVs derived from melanoma cells disrupted haematopoiesis, mirroring characteristics observed during the immune escape phase of tumour development. For instance, mice injected with EVs exhibited EMH, reduced bone marrow cellularity, medullary expansion of MDSCs, and the development of anaemia. These observations paralleled those found in mice with advanced melanoma. In accordance with these findings, it has been demonstrated that systemic treatment of both naïve mice and mice undergoing hematopoietic reconstitution with melanoma cell‐derived EVs led to significant alterations in the cellular compositions of primary and secondary immune organs, namely the bone marrow, spleen, and lymph nodes, respectively (Du et al., 2022). Overall, these alterations indicated the development of an immunosuppressive state characterized by decreased frequencies of CD8 T cells and NK cells, expansion of G‐MDSCs, and upregulation of PD‐1/PD‐L1 immune regulatory molecules in CD4 T cells and myeloid cell subsets (Du et al., 2022). Collectively, these findings suggest that as intracellular communicators, tEVs can play a significant role in promoting immunosuppressive activities and dysregulation of haematopoiesis. However, further studies are required to fully elucidate the underlying mechanisms. Subsequent in vivo experiments unveiled that the factors carried by the EVs, which were accountable for the disruption of haematopoiesis, displayed sensitivity to heat. In other words, short exposure to a temperature of 100°C was sufficient to nullify the tEV‐induced expansion of MDSCs in the BM cell culture. This finding strongly implies that one or potentially several components associated with tEVs could be responsible for the induction of haematopoiesis dysregulation. In this context, accumulating evidence suggests that the presence of the most abundant angiogenic factor, VEGF on tEVs can play a pivotal role. For instance, continuous VEGF infusion has been shown to inhibit the development of dendritic cell while potently increasing the production of MDSCs cells and B cells (Gabrilovich et al., 1998). Further studies have uncovered that VEGF induces the accumulation of MDSCs through its receptor VEGFR2, while utilizing VEGFR1 to facilitate the expansion of B cells (Huang et al., 2007). Additionally, it has been demonstrated that VEGF induces erythropoietin production in the kidney, liver, and spleen in a hypoxia‐independent manner, leading to the development of extramedullary erythropoiesis (Greenwald et al., 2019). Consistent with these observations, our ex vivo studies have shown that EVs derived from melanoma cells could independently induce the expansion of bone marrow MDSCs and B cells while simultaneously reducing the frequency of cells belonging to the erythropoietic lineage. More importantly, the outcome of our ex vivo study indicated that the impact of tEVs was severely diminished when they were exposed to a temperature of 56°C and when VEGFR on the BM cells was blocked. These findings substantiate the direct role of VEGF as a protein constituent associated with tEVs in the induction of dysregulated haematopoiesis.

Our findings revealed that, in addition to VEGF, the levels of ADAMST1, Tissue Factor and OPN are also significantly enriched as associated angiogenic factors in tEVs. The results of our ex vivo studies showed that blocking of the activities of these factors exerted minor impact on the haematopoiesis dysregulation caused by tEVs. However, further investigations are required to fully elucidate the role these tEV‐associated factors alone or in combination in the immune escape phase of cancer‐induced immunosuppression.

## 5 CONCLUSION

Our study presents evidence supporting the crucial role of tEVs as a form of indirect cell‐to‐cell communicators in causing haematopoiesis dysregulation, a phenomenon that occurs during immune escape. As we have shown, tEVs carrying potent angiogenic factors, particularly VEGF, can reprogram the process of haematopoiesis in both primary and secondary immune organs. Therefore, it is essential to consider strategies that target tEV production to suppress the immune escape phase and prevent dysregulation of haematopoiesis during cancer development.

## AUTHOR CONTRIBUTIONS

**Doste R. Mamand**: Conceptualization; data curation; formal analysis; funding acquisition; investigation; methodology; resources; supervision; visualization; writing—original draft; writing—review and editing. **Safa Bazaz**: Data curation; formal analysis; investigation; methodology; validation; visualization; writing—review and editing. **Dara K. Mohammad**: Formal analysis; investigation; methodology; writing—review and editing. **Xiuming Liang**: Investigation; methodology; writing—review and editing. **Svetlana Pavlova**: Investigation; methodology; writing—review and editing. **Carsten Mim**: Investigation; writing—review and editing. **Susanne Gabrielsson**: Formal analysis; investigation; methodology; writing—review and editing. **Joel Z. Nordin**: Formal analysis; investigation; methodology; writing—review and editing. **Oscar P. B. Wiklander**: Conceptualization; data curation; formal analysis; investigation; methodology; supervision; visualization; writing—review and editing. **Manuchehr Abedi‐Valugerdi**: Conceptualization; data curation; formal analysis; funding acquisition; investigation; methodology; project administration; resources; supervision; writing—original draft; writing—review and editing. **Samir EL‐Andaloussi**: Conceptualization; data curation; formal analysis; funding acquisition; investigation; methodology; project administration; resources; supervision; writing—review and editing.

## CONFLICT OF INTEREST STATEMENT

Samir El‐Andaloussi and Joel Z. Nordin are the founder of and consultant for Evox Therapeutics. Oscar P. B. Wiklander, Samir El‐Andaloussi and Joel Z. Nordin have stock interest in Evox Therapeutics. Susanne Gabrielsson has a patent on B cell‐derived exosomes in immune therapy and is part of the Scientific Advisory Board of Anjarium Biosciences. The other authors declare no competing interests.

## Supporting information

Supporting Information
