# Supplementary material for: Extracellular vesicles originating from melanoma cells promote dysregulation in haematopoiesis as a component of cancer immunoediting
Source: J Extracell Vesicles. 2024 Jun 29;13(7):e12471. doi: 10.1002/jev2.12471 (PMC11214607; doi:10.1002/jev2.12471)
Supplement: Supplementary file 1 — Supporting Information [file JEV2-13-e12471-s001.docx]

**Extracellular vesicles originating from melanoma cells promote dysregulation in hematopoiesis as a component of cancer immunoediting**

**Running title: Induction of hematopoiesis dysregulation by melanoma cell-derived EVs**

Doste R. Mamand^1,2&,^ Safa Bazaz^1,2^, Dara K. Mohammad^3,4^, Xiuming Liang^1,2^, Svetlana Pavlova^1,2^, Carsten Mim^6^, Susanne Gabrielsson^7^, Joel Z. Nordin^1,5^, Oscar P. B. Wiklander^1,2**,^ Manuchehr Abedi-Valugerdi^1,2**&,^ Samir EL-Andaloussi^1,2**^

^1^Biomolecular and Cellular Medicine (BMC), Department of Laboratory Medicine , Karolinska Institutet, Huddinge, Sweden

^2^Department of Cellular Therapy and Allogeneic Stem Cell Transplantation (CAST), Karolinska University Hospital Huddinge and Karolinska Comprehensive Cancer Center, Stockholm, Sweden

^3^Center for Hematology and Regenerative Medicine (HERM), Department of Medicine Huddinge, Karolinska Institutet, SE-141 83 Stockholm, Sweden

^4^College of Agricultural Engineering Sciences, Salahaddin University-Erbil, Kurdistan Region, Erbil 44002, Iraq

^5^Department of Clinical Immunology and Transfusion Medicine (KITM), Karolinska University Hospital, 141 86 Stockholm, Sweden

^6^Department of Protein Science, KTH Royal Institute of Technology

^7^Division of Immunology and Allergy, Department of Medicine Solna, Karolinska Institutet, Solna, Sweden

^**^These authors have contributed equally

^&^Correspondences

^**, &^Manuchehr Abedi-Valugerdi, PhD, Biomolecular and Cellular Medicine, Clinical Research Center, Novum, Department of Laboratory Medicine , Karolinska Institutet, Huddinge, 141 86 Stockholm, Sweden, E-mail address: manuchehr.abedi-valugerdi@ki.se.

^&^Doste Mamand, MSc, Biomolecular and Cellular Medicine, Clinical Research Center, Novum, Department of Laboratory Medicine , Karolinska Institutet, Huddinge, 141 86 Stockholm, Sweden, E-mail address: doste.rashid.mamand@ki.se.


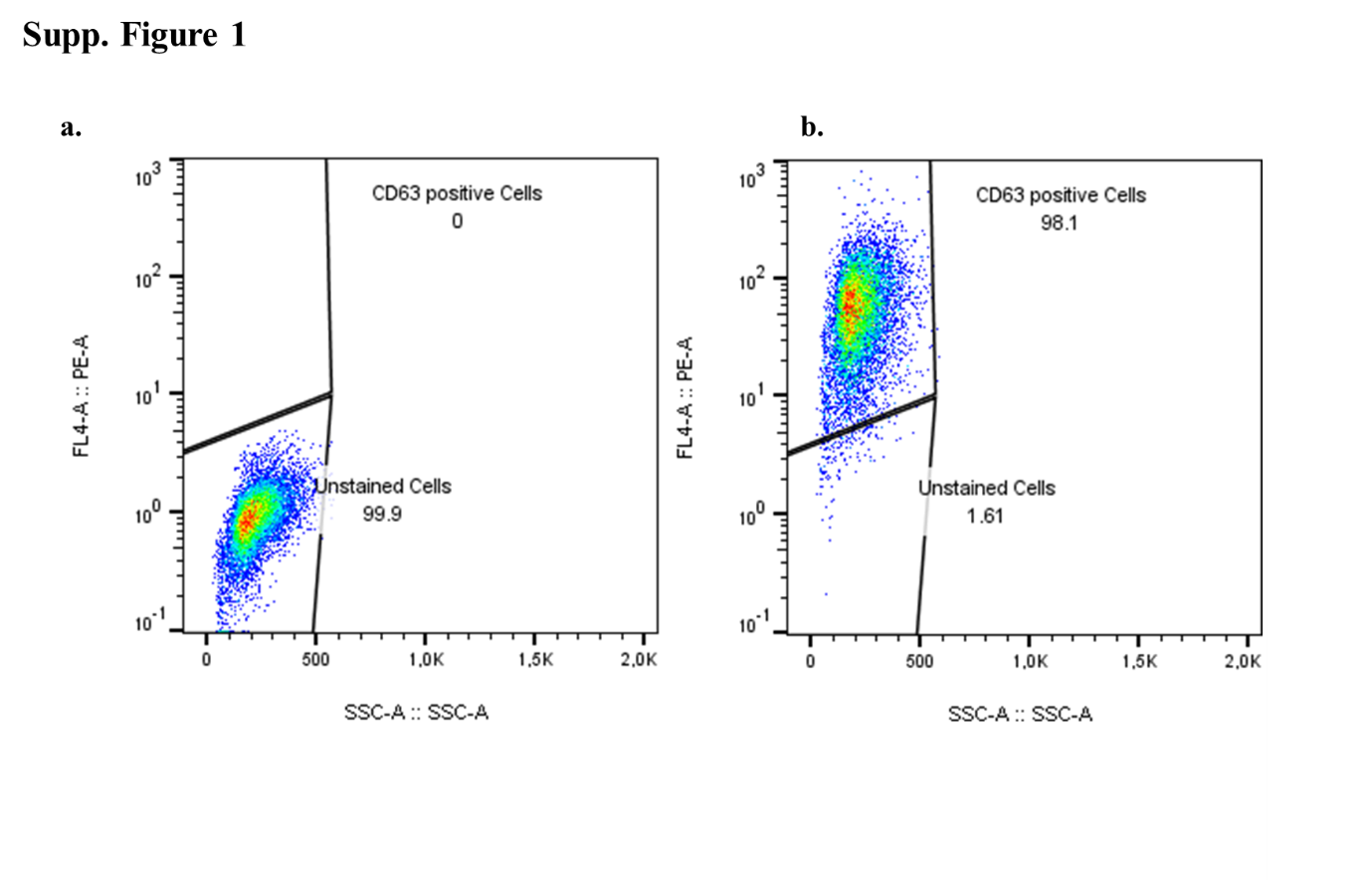


Supplementary Figure 1. Detecting tetraspanin CD63 on B16F10 cells using flow cytometry. a. unstained B16F10 cells. a. Tetraspanin-CD63-labelled Phycoerythrin (PE) anti-mouse was identified on B16f10 cells in accordance with the site scatter signal (SSC).


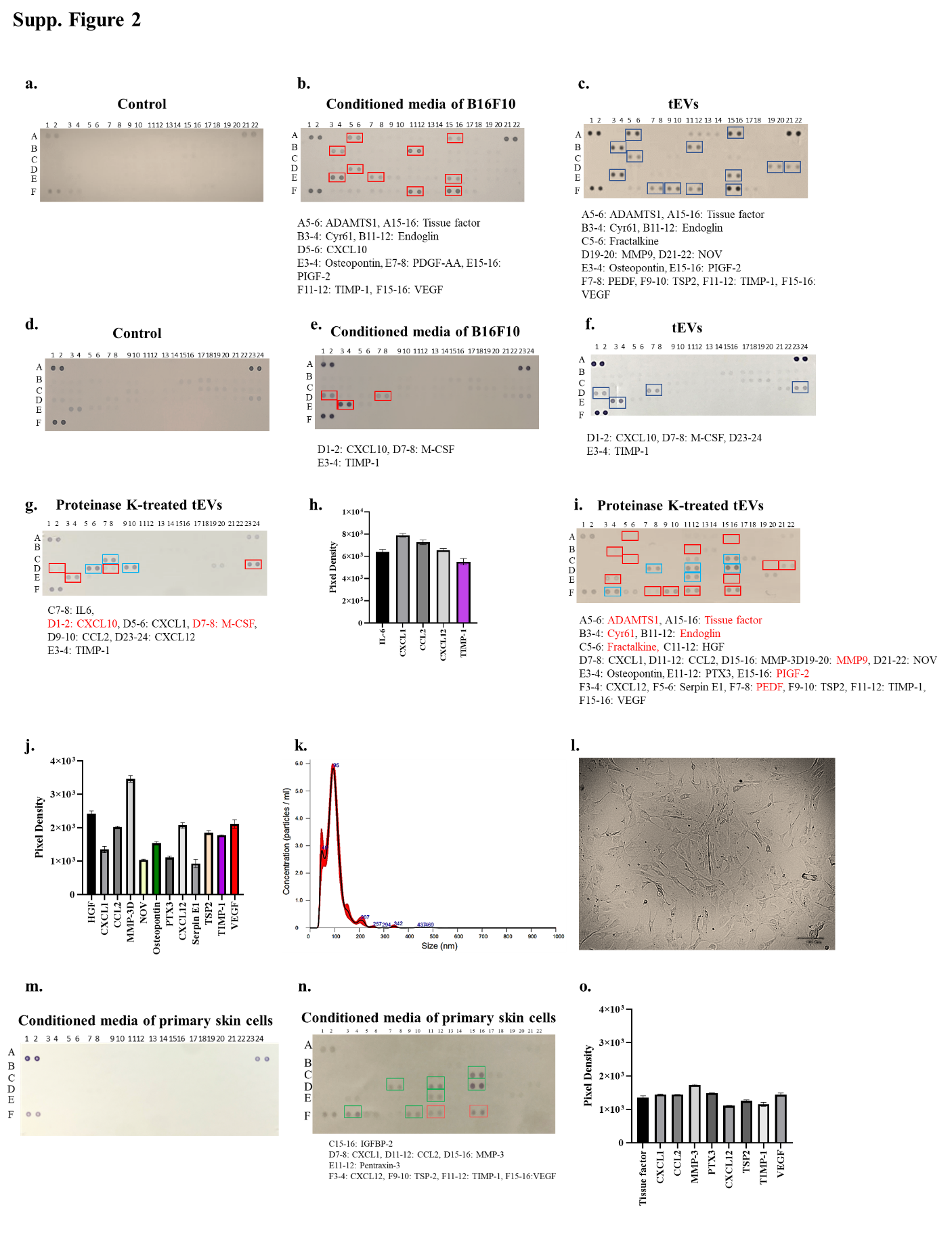


Supplementary Figure 2. Characterization of tEVs isolated from B16F10 cells and proteomic profiling. a. The control membrane incubated with 1 ml of DMEM complete media. b. The different expressions of 10 angiogenic factors on the membrane incubated with 1 ml of B16F10 supernatant for 3 days. c. Analysis of the membrane shows the different expression of 13 angiogenic factors on the membrane incubated with 2 x 10^11^ tEVs. d. The control membrane that was treated with 1 ml of DMEM complete media. e. The different expression of three chemokines on the membrane that was treated with 1 ml of B16F10 supernatant for three days. f. The membrane shows the different expression of four cytokines on the membrane that was treated with 2 x 10^11^ tEVs. g. Proteinase K treated tEVs shows the different expression of chemokines on the membrane that was treated with 1 ml of fibroblast primary cells supernatant for three days. h. Histogram profiles for selected analytes were created by calculating the mean spot pixel densities with standard imaging software. i. Proteinase K treated tEVs shows the different expression of angiogenesis on the membrane that was treated with 1 ml of fibroblast primary cells supernatant for three days. j. Histogram profiles for selected analytes were created by calculating the mean spot pixel densities with standard imaging software. k. Nanoparticle analysis of Proteinase K treated tEVs demonstrates single-peak distribution a mean size of 95 nm in diameter. l. microscopic image of fibroblast primary cells. m. the different expression of chemokines on the membrane that was treated with 1 ml of fibroblast primary cells supernatant for three days. n. The different expression of angiogenesis on the membrane that was treated with 1 ml of fibroblast primary cells supernatant for three days. o. Histogram profiles for selected analytes were created by calculating the mean spot pixel densities with standard imaging software. h, i and o) The data are shown as means (± SD, n = 2). e, g) The experiment has been repeated similarly twice.


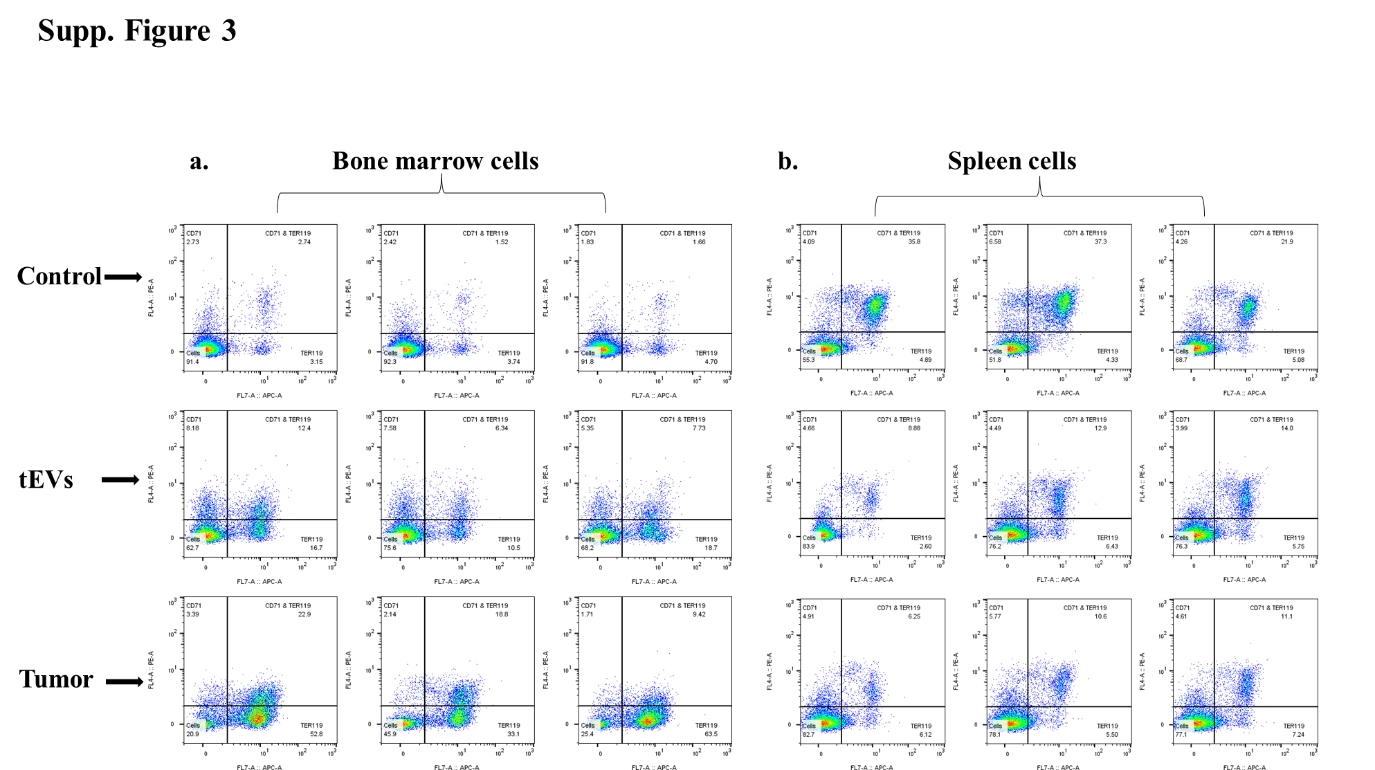


Supplementary Figure 3. Representative flow cytometric examination of bone marrow and spleen cells. a. Early EPCs (CD71+), late EPCs (CD71+/TER119+), erythrocytes (TER119+), in control, tEVs, and tumor-bearing mice groups. b. Splenic early EPCs (CD71+), late EPCs (CD71+/TER119+), and erythrocytes (TER119+) in control, tEVs, and tumor-bearing mice groups. A flow cytometric examination was run on three biological replicates using TER119-APC-conjugated Abs (x-axis) and CD71-PE-conjugated Abs (y-axis).


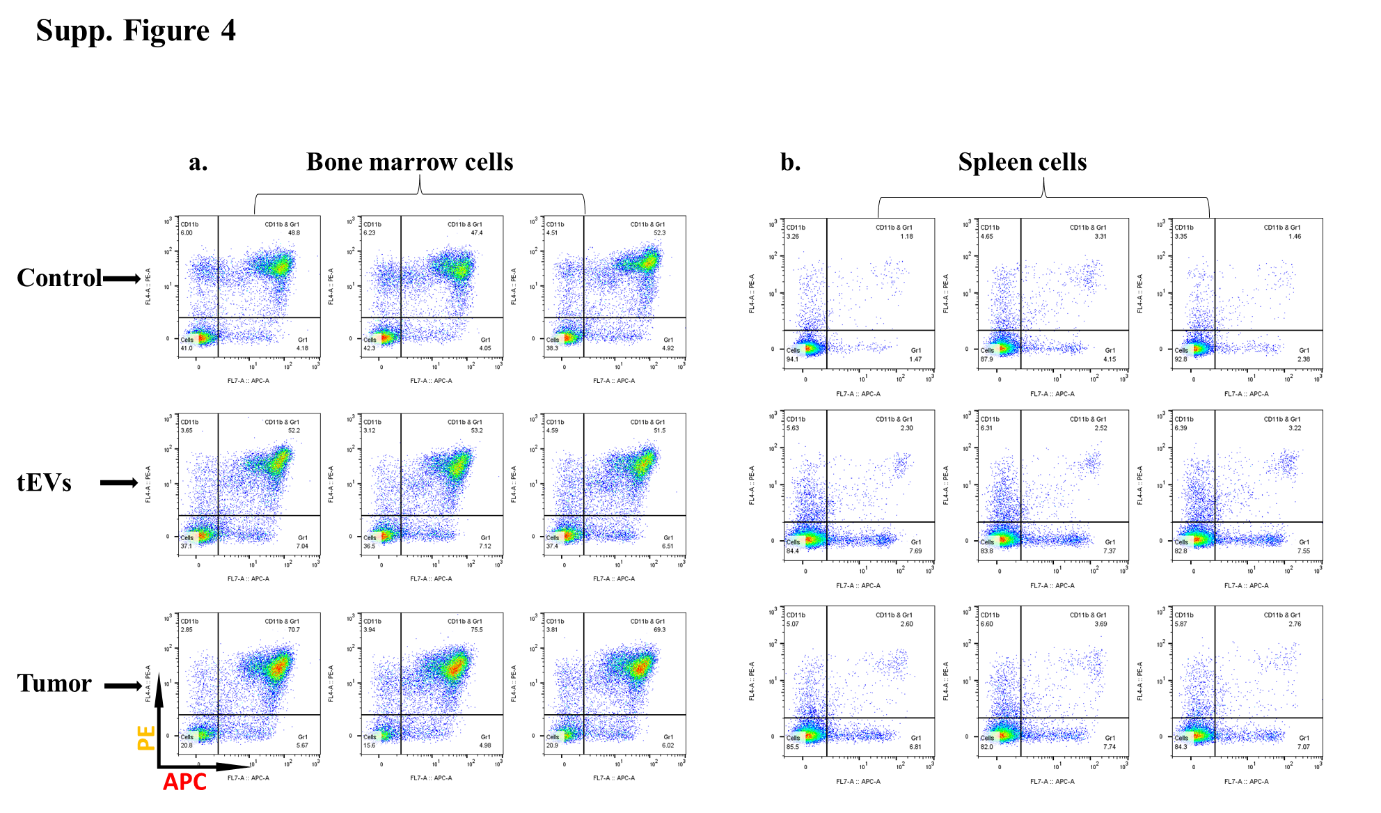


Supplementary Figure 4. Representative flow cytometric examination of bone marrow and spleen cells. a. Bone marrow granulocytes (Gr1+), macrophages (CD11b+), and MDSCs (Gr1+/CD11b) in the control, tEVs, and tumor-bearing mice groups. b. Splenic granulocytes (Gr1+), macrophages (CD11b+), and MDSCs (Gr1+/CD11b) in the control, tEVs, and tumor-bearing mice groups. A flow cytometric examination was run on three biological replicates using Gr1-APC-conjugated Abs (x-axis) and CD11b-PE-conjugated Abs (y-axis).


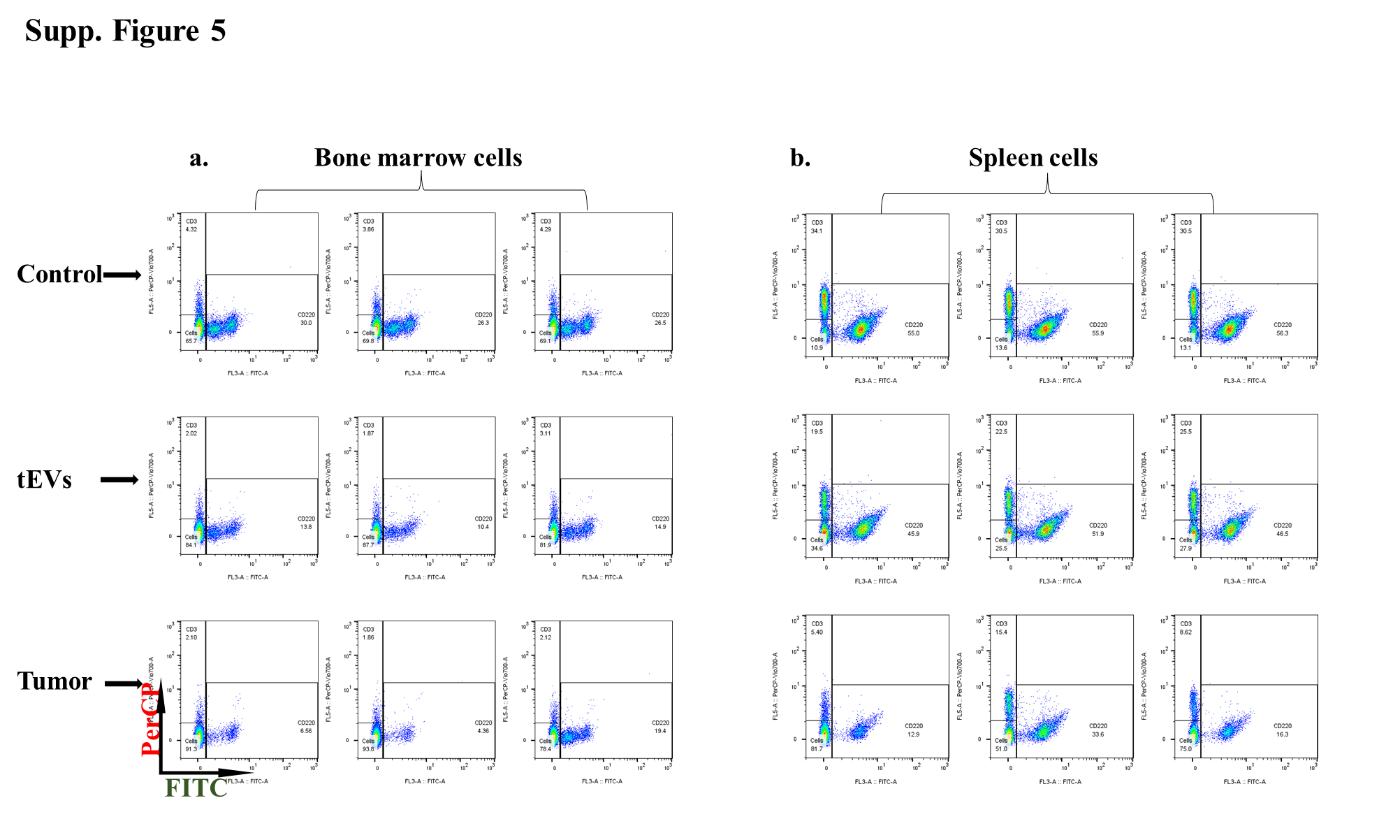


Supplementary Figure 5. Representative flow cytometric examination of bone marrow and spleen cells. a. Bone marrow B cells (CD220+) and T cells (CD3+) in the control, tEVs, and tumor-bearing mice groups. b. Splenic B cells (CD220+) and T cells (CD3+) in the control, tEVs, and tumor-bearing mice groups. A flow cytometric examination was run on three biological replicates using B220-APC-conjugated Abs (x-axis) and CD3-PrePCP-conjugated Abs (y-axis).


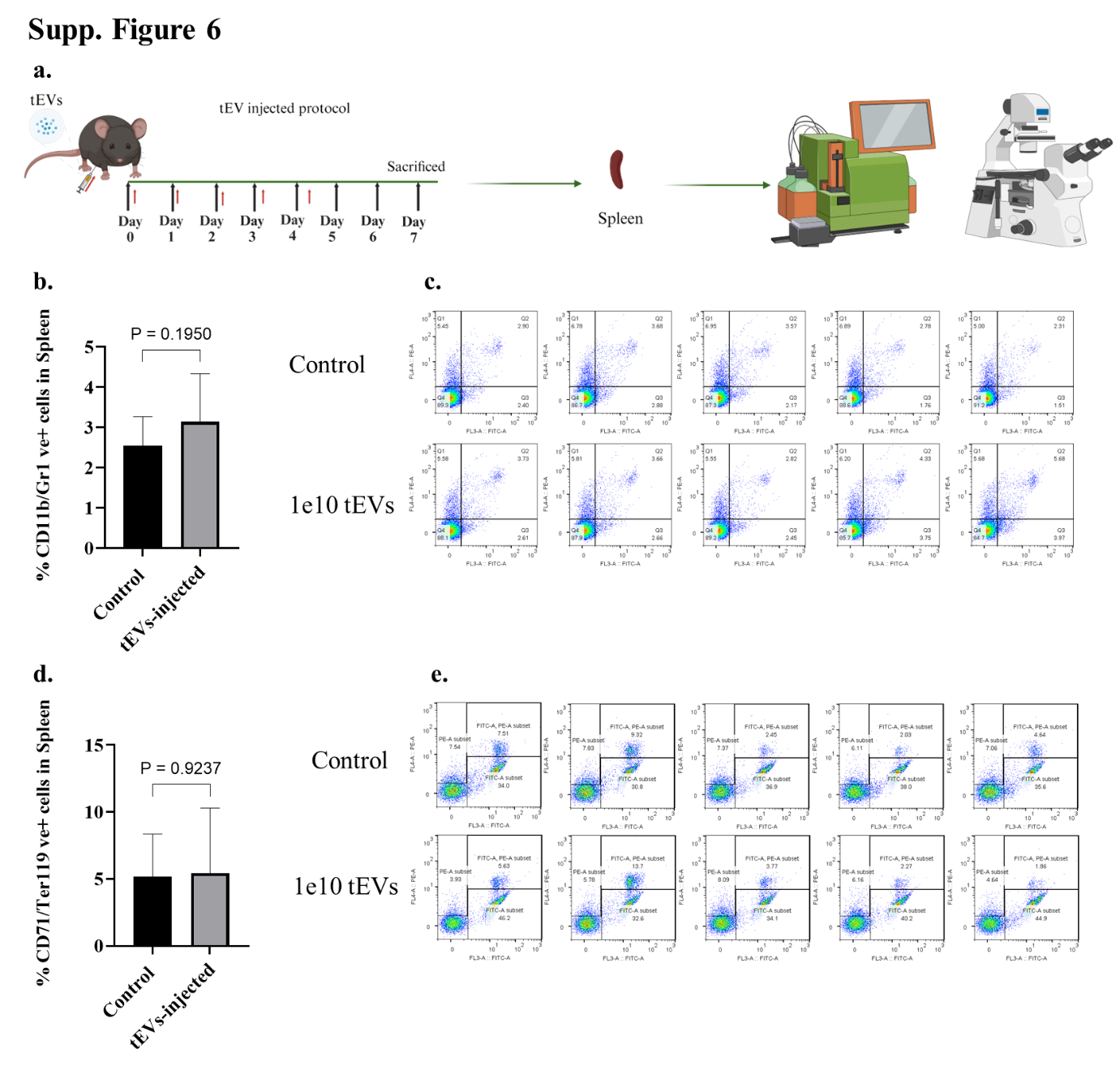


Supplementary Figure 6. Induction of immunosuppressive EPCs and MDSCs in the spleen by low dose of tEVs. a. Schematic illustration of tEVs workflow in vivo using female C57BL/6N mice that were either un-injected (control, n = 5) or i.p. injected with 1x10^10^ tEVs every day for 5 days (n = 5). b. c. Flow cytometry was employed to assess the frequency of the distinct cell types within the spleens (b). Representative flow cytometric examination of splenic granulocytes (Gr1+), macrophages (CD11b+), MDSCs (Gr1+/CD11b+), early EPCs (CD71+), late EPCs (CD71+/TER119+), erythrocytes (TER119+). d. e. Flow cytometry was employed to assess the frequency of the distinct cell types within the spleens (b). Representative flow cytometric examination of splenic early EPCs (CD71+), late EPCs (CD71+/TER119+), erythrocytes (TER119+). The data are presented as means (± SD, n = 5). Differences between the groups were analyzed statistically using the Mann-Whitney U test.


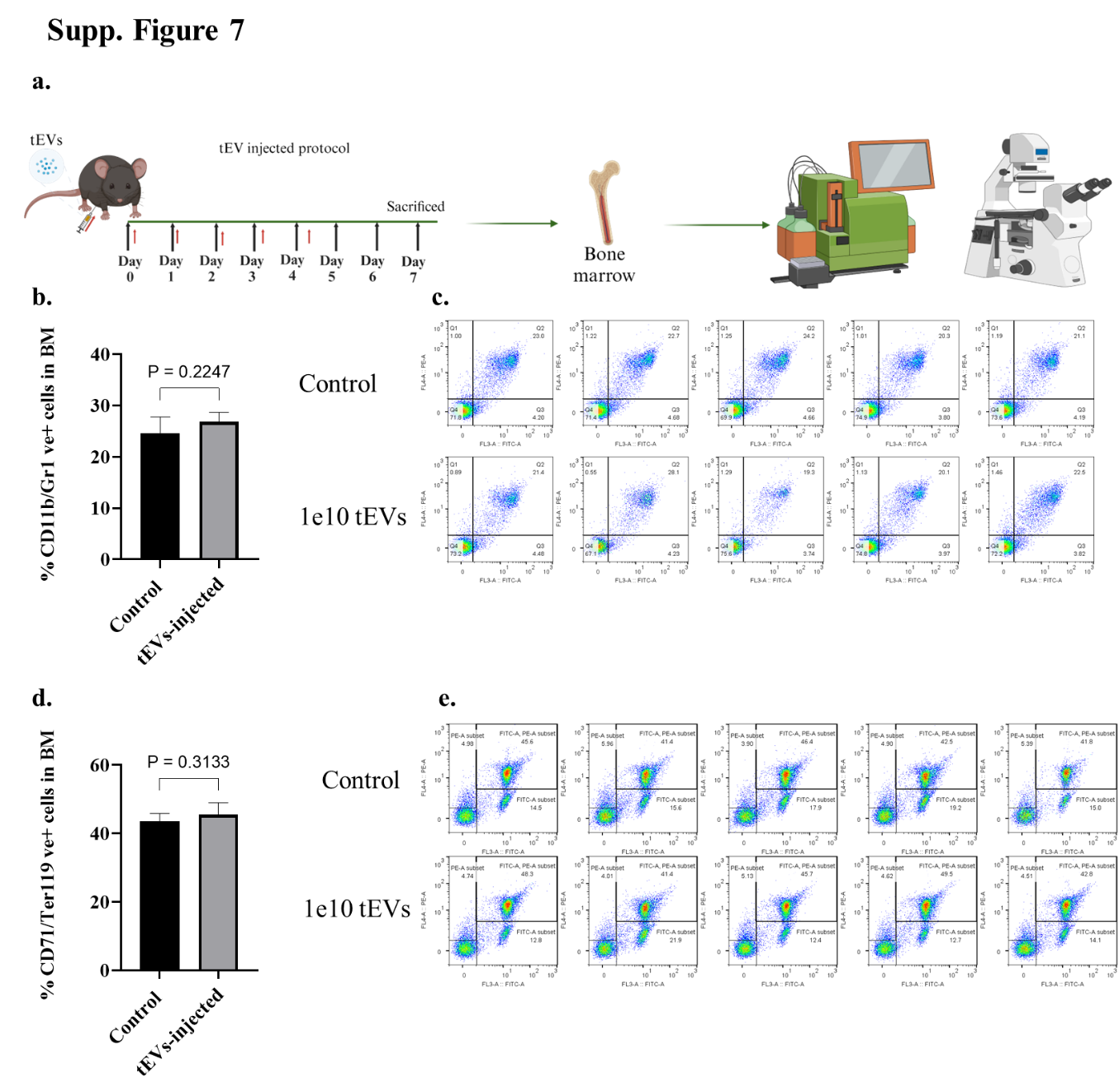


Supplementary Figure 7. Induction of immunosuppressive EPCs and MDSCs in the bone marrow by low dose of tEVs. a. Schematic illustration of tEVs workflow in vivo using female C57BL/6N mice that were either un-injected (control, n = 5) or i.p. injected with 1x10^10^ tEVs every day for 5 days (n = 5). b. c. Flow cytometry was employed to assess the frequency of the distinct cell types within the spleens (b). Representative flow cytometric examination of splenic granulocytes (Gr1+), macrophages (CD11b+), MDSCs (Gr1+/CD11b+), early EPCs (CD71+), late EPCs (CD71+/TER119+), erythrocytes (TER119+). d. e. Flow cytometry was employed to assess the frequency of the distinct cell types within the spleens (b). Representative flow cytometric examination of splenic early EPCs (CD71+), late EPCs (CD71+/TER119+), erythrocytes (TER119+). The data are presented as means (± SD, n = 5). Differences between the groups were analyzed statistically using the Mann-Whitney U test.


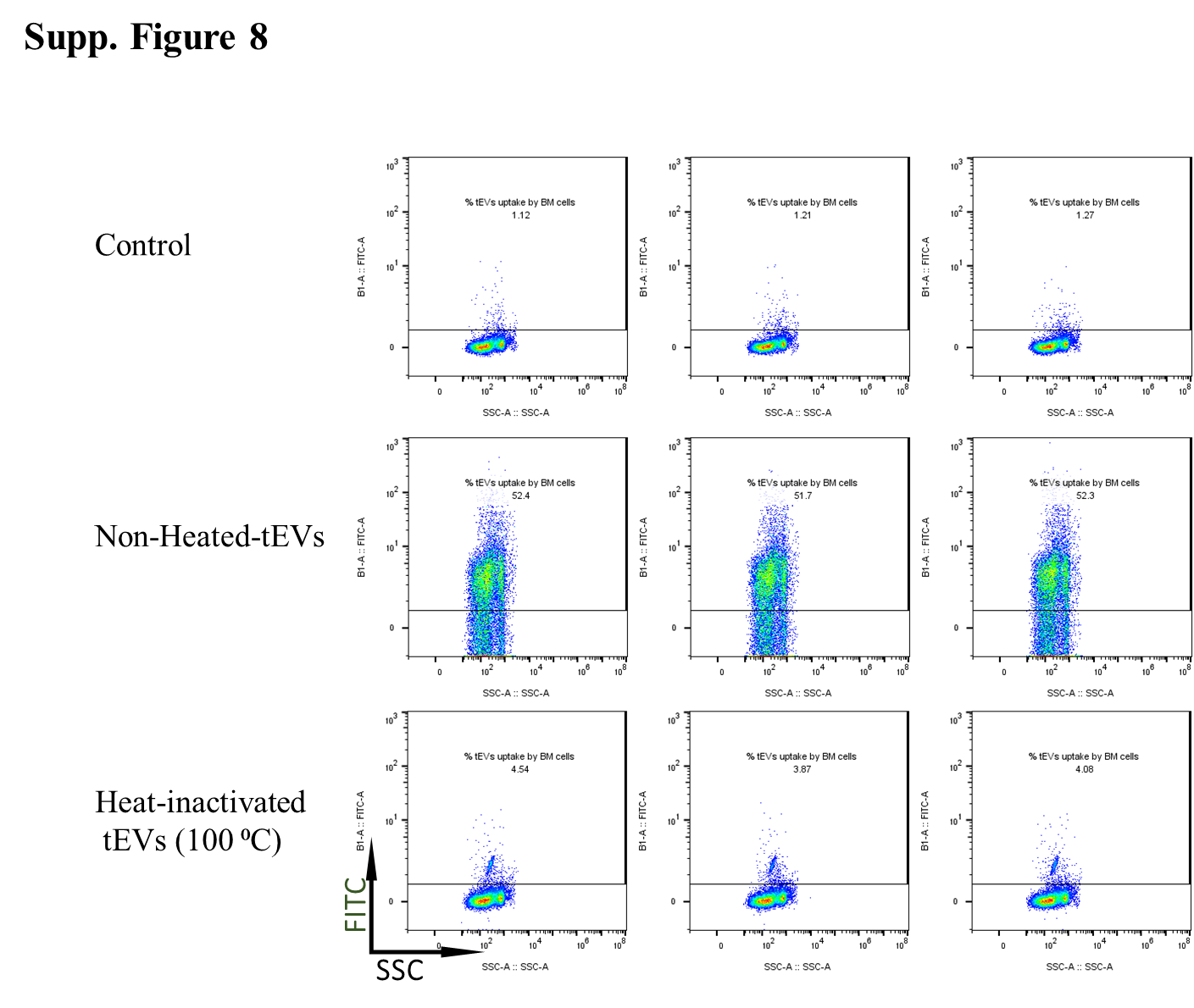


Supplementary Figure 8. Cellular uptake of fluorescent tEVs. Flow cytometry was employed to assess the internalization of 5x10^9^ fluorescent tEVs (mNG-tEVs) by bone marrow cells after 4 hours.


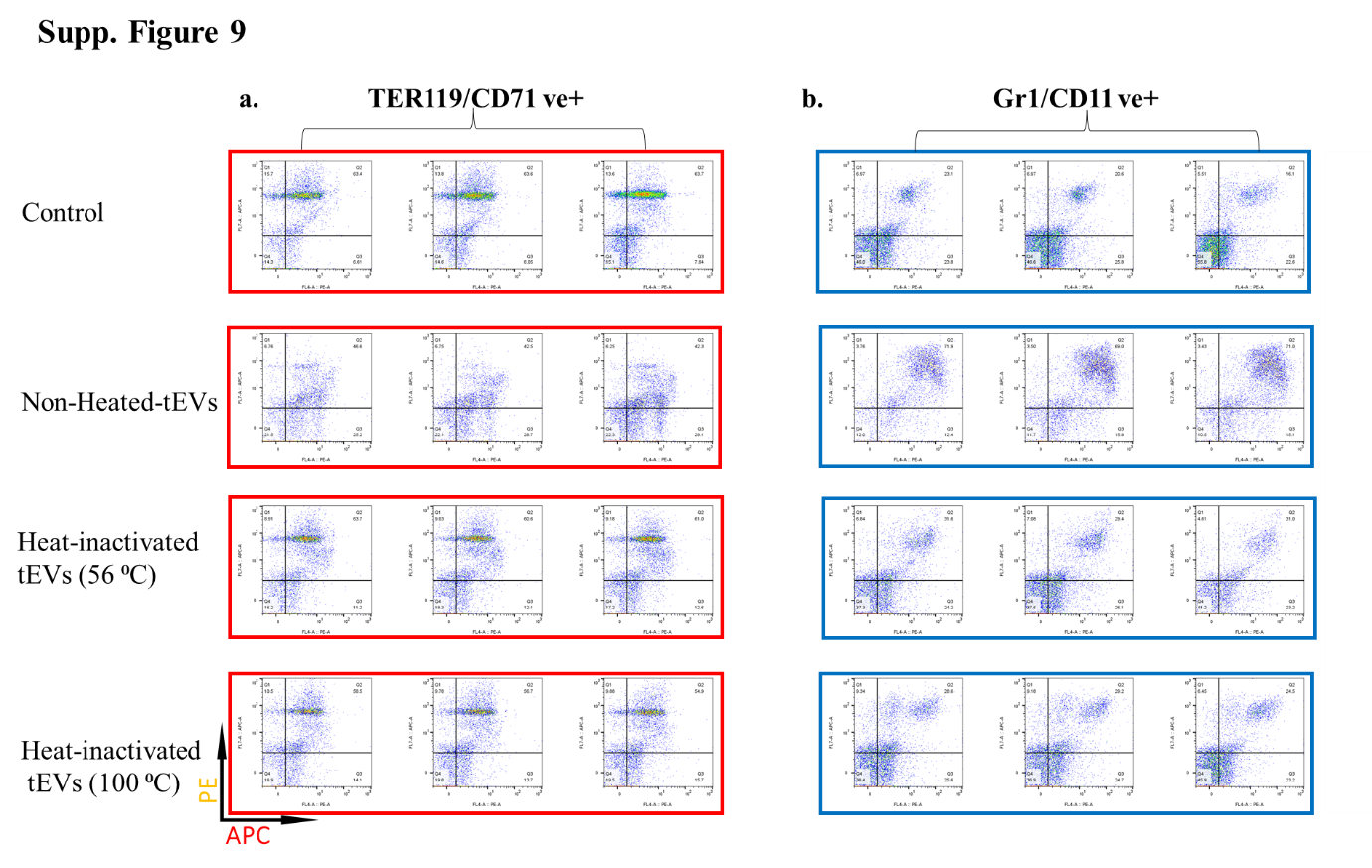


Supplementary Figure 9. Representative flow cytometric examination of bone marrow cells. a. Early EPCs (CD71+), late EPCs (CD71+/TER119+), erythrocytes (TER119+), in control, non-heated tEVs, heated tEVs at 56 ^o^C, and heated tEVs at 100 ^o^C. b. Bone marrow granulocytes (Gr1+), macrophages (CD11b+), and MDSCs (Gr1+/CD11b) in control, non-heated tEVs, heated tEVs at 56 ^o^C, and heated tEVs at 100 ^o^C. A flow cytometric examination was run on three biological replicates using TER119-APC-conjugated Abs (x-axis) and CD71-PE-conjugated Abs (y-axis) red panel, Gr1-APC-conjugated Abs (x-axis) and CD11b-PE-conjugated Abs (y-axis) blue panel.


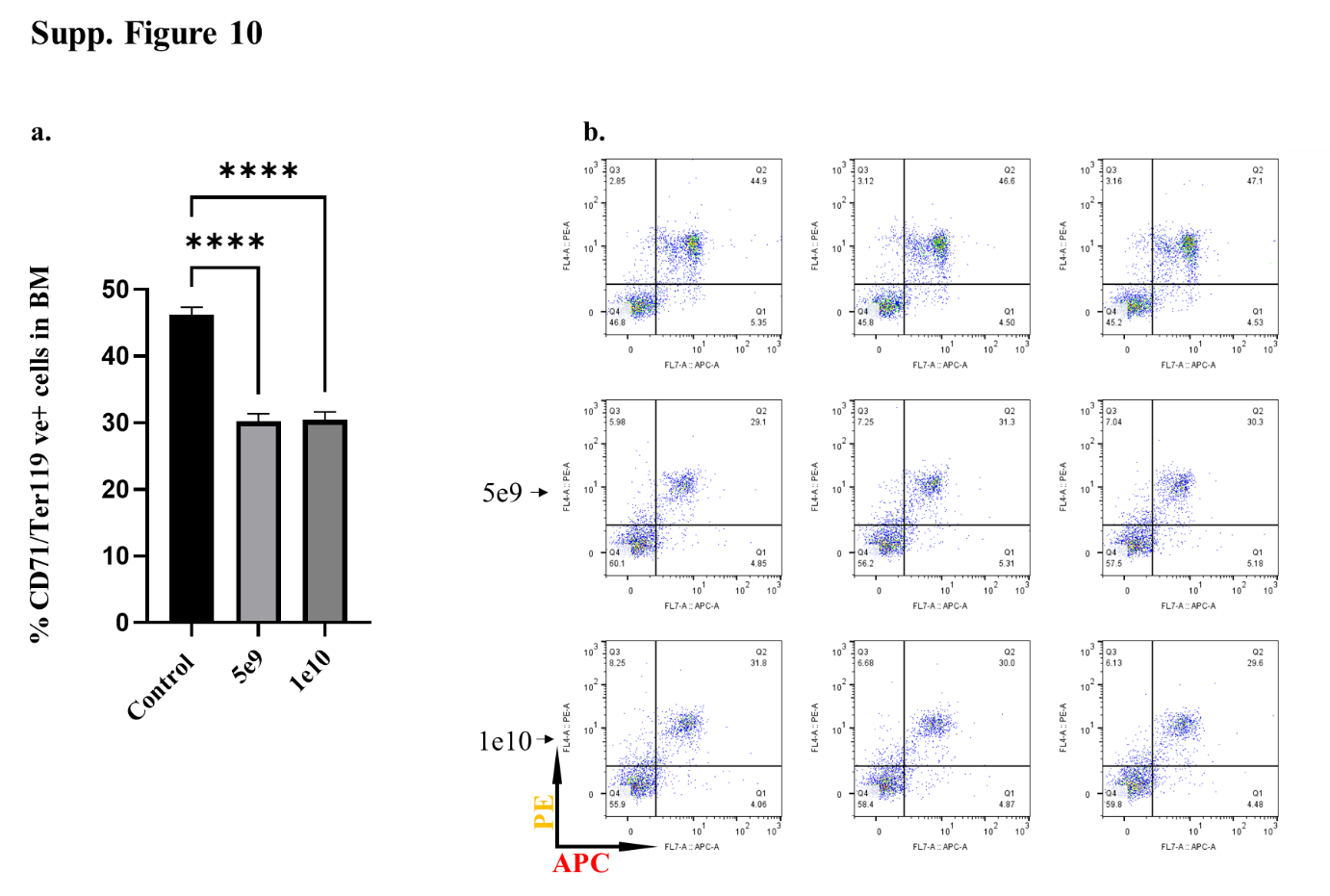


Supplementary Figure 10. Different tEVs doses have an effect on bone marrow cells in ex-vivo. a. Frequency of early EPCs (CD71+), late EPCs (CD71+/TER119+), and erythrocytes (TER119+), in control, 5 x 10^9^ tEVs, and 1 x 10^10^ tEVs. b. Representative flow cytometric examination of bone marrow cells of early EPCs (CD71+), late EPCs (CD71+/TER119+), erythrocytes (TER119+), in control, 5 X10^9^ tEVs, and 1 X10^10^ tEVs, using TER119-APC-conjugated Abs (x-axis) and CD71-PE-conjugated Abs (y-axis). The data are presented as means (± SD, n = 3). Differences between the groups were analyzed statistically employing the Mann-Whitney U test (****P<0.001).


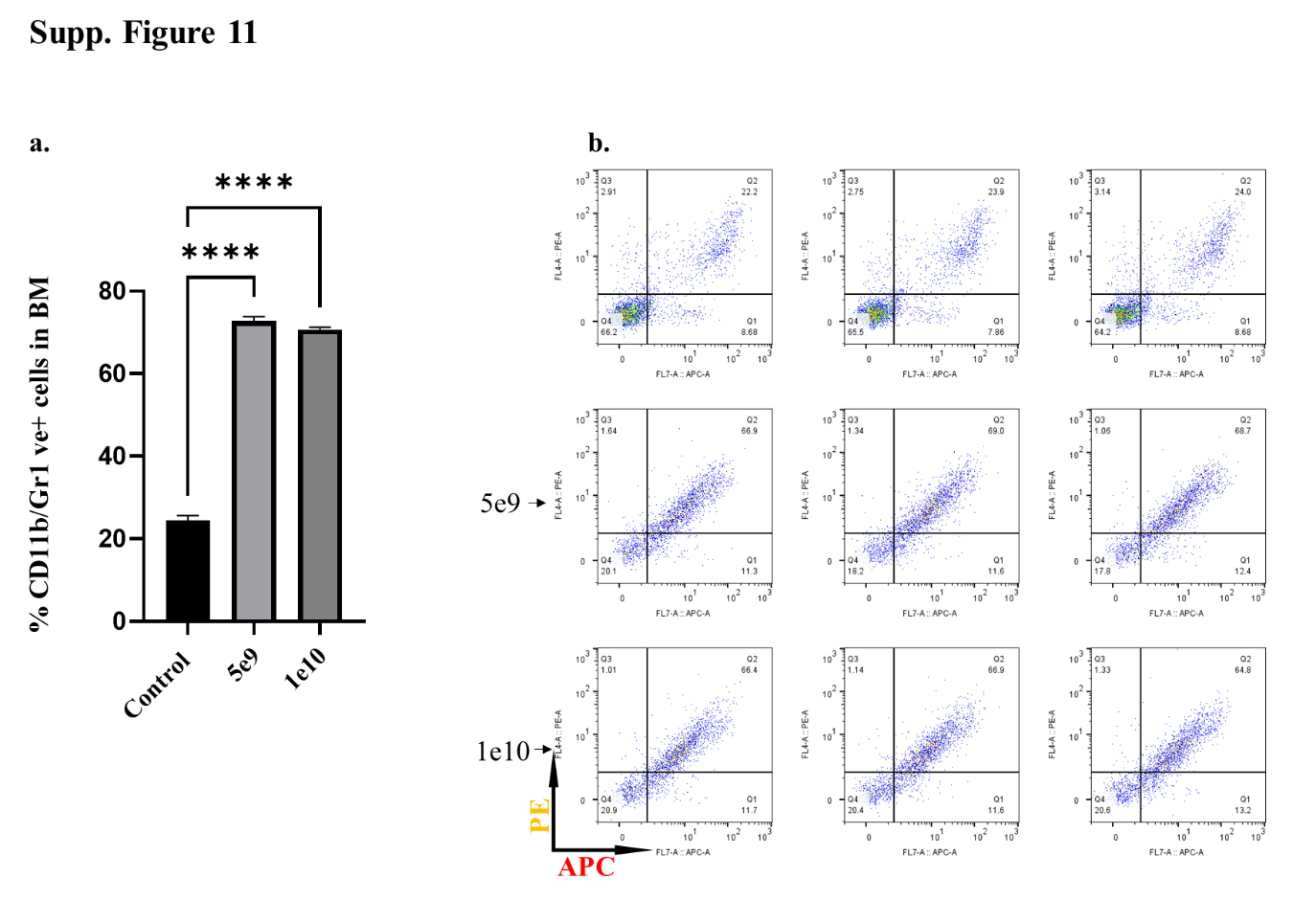


Supplementary Figure 11. Different tEVs doses have an effect on bone marrow cells in ex-vivo. a. Bone marrow granulocytes (Gr1+), macrophages (CD11b+), and MDSCs (Gr1+/CD11b), in control, 5 X10^9^ tEVs, and 1 x10^10^ tEVs. b. Representative flow cytometric examination of bone marrow cells, granulocytes (Gr1+), macrophages (CD11b+), and MDSCs (Gr1+/CD11b) in control, 5 X10^9^ tEVs, and 1 x10^10^ tEVs, using Gr1-APC-conjugated Abs (x-axis) and CD11b-PE-conjugated Abs (y-axis). The data are presented as means (± SD, n = 3). Differences between the groups were analyzed statistically employing the Mann-Whitney U test (****P<0.001).


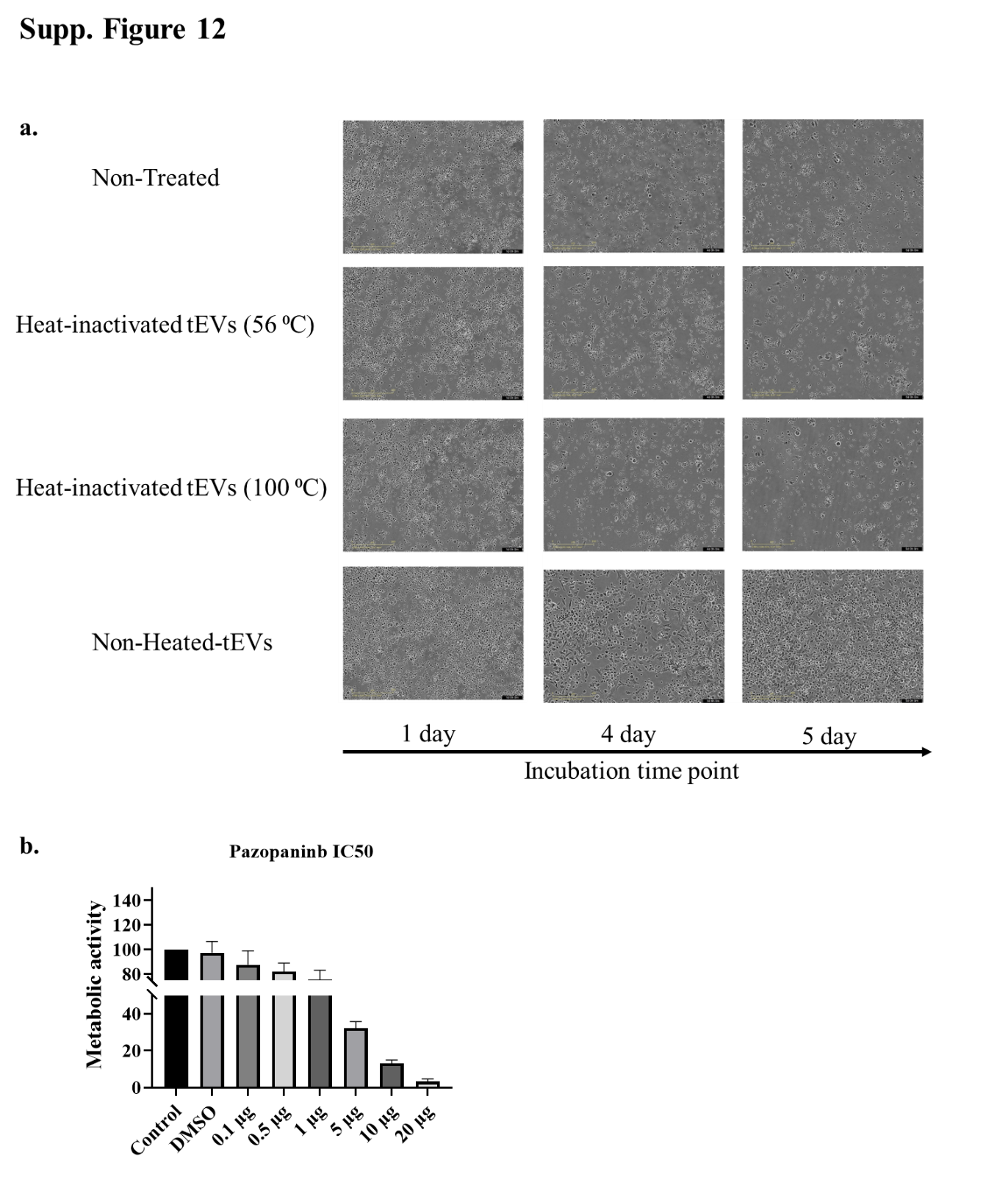


Supplementary Figure 12. Representative IncuCyte live-cell imaging of bone marrow cells at different time points BM cells were co-cultured with tEVs: non-heated 5x10^9^, 5x10^9^ heated at 56 ^o^ C, and 5x10^9^ heated at 100 ^o^ C. Data analysis was performed on real live-cell images. b. Pazopanib IC50 on bone marrow cells after 3 days of incubation. The data are presented as means (± SD, n = 3).
